# Urinary metabolomics provide insights into coronary artery disease in individuals with type 1 diabetes

**DOI:** 10.1186/s12933-024-02512-8

**Published:** 2024-11-26

**Authors:** Anni A. Antikainen, Stefan Mutter, Valma Harjutsalo, Lena M. Thorn, Per-Henrik Groop, Niina Sandholm, the FinnDiane Study Group

**Affiliations:** 1 Folkhälsan Institute of Genetics, Folkhälsan Research Center, 00290 Helsinki, Finland; 2 Department of Nephrology, University of Helsinki and Helsinki University Hospital, 00290 Helsinki, Finland; 3 Research Program for Clinical and Molecular Metabolism, Faculty of Medicine, University of Helsinki, 00290 Helsinki, Finland; 4 Department of General Practice and Primary Health Care, University of Helsinki and Helsinki University Hospital, 00014 Helsinki, Finland; 5 Department of Diabetes, Central Clinical School, Monash University, Melbourne, VIC Australia; 6 Baker Heart and Diabetes Institute, Melbourne, VIC Australia

**Keywords:** Type 1 diabetes, Coronary artery disease, Cardiac complication, Metabolomics, Urine, Oxidative stress, Survival modeling, Network analysis, Machine learning

## Abstract

**Background:**

Type 1 diabetes increases the risk of coronary artery disease (CAD). High-throughput metabolomics may be utilized to identify metabolites associated with disease, thus, providing insight into disease pathophysiology, and serving as predictive markers in clinical practice. Urine is less tightly regulated than blood, and therefore, may enable earlier discovery of disease-associated markers. We studied urine metabolomics in relation to incident CAD in individuals with type 1 diabetes.

**Methods:**

We prospectively studied CAD in 2501 adults with type 1 diabetes from the Finnish Diabetic Nephropathy Study. 209 participants experienced incident CAD within the 10-year follow-up. We analyzed the baseline urine samples with a high-throughput targeted urine metabolomics platform, which yielded 54 metabolites. With the data, we performed metabolome-wide survival analyses, correlation network analyses, and metabolomic state profiling for prediction of incident CAD.

**Results:**

Urinary 3-hydroxyisobutyrate was associated with decreased 10-year incident CAD, which according to the network analysis, likely reflects younger age and improved kidney function. Urinary xanthosine was associated with 10-year incident CAD. In the network analysis, xanthosine correlated with baseline urinary allantoin, which is a marker of oxidative stress. In addition, urinary trans-aconitate and 4-deoxythreonate were associated with decreased 5-year incident CAD. Metabolomic state profiling supported the usage of CAD-associated urinary metabolites to improve prediction accuracy, especially during shorter follow-up. Furthermore, urinary trans-aconitate and 4-deoxythreonate were associated with decreased 5-year incident CAD. The network analysis further suggested glomerular filtration rate to influence the urinary metabolome differently between individuals with and without future CAD.

**Conclusions:**

We have performed the first high-throughput urinary metabolomics analysis on CAD in individuals with type 1 diabetes and found xanthosine, 3-hydroxyisobutyrate, trans-aconitate, and 4-deoxythreonate to be associated with incident CAD. In addition, metabolomic state profiling improved prediction of incident CAD.

**Graphical abstract:**

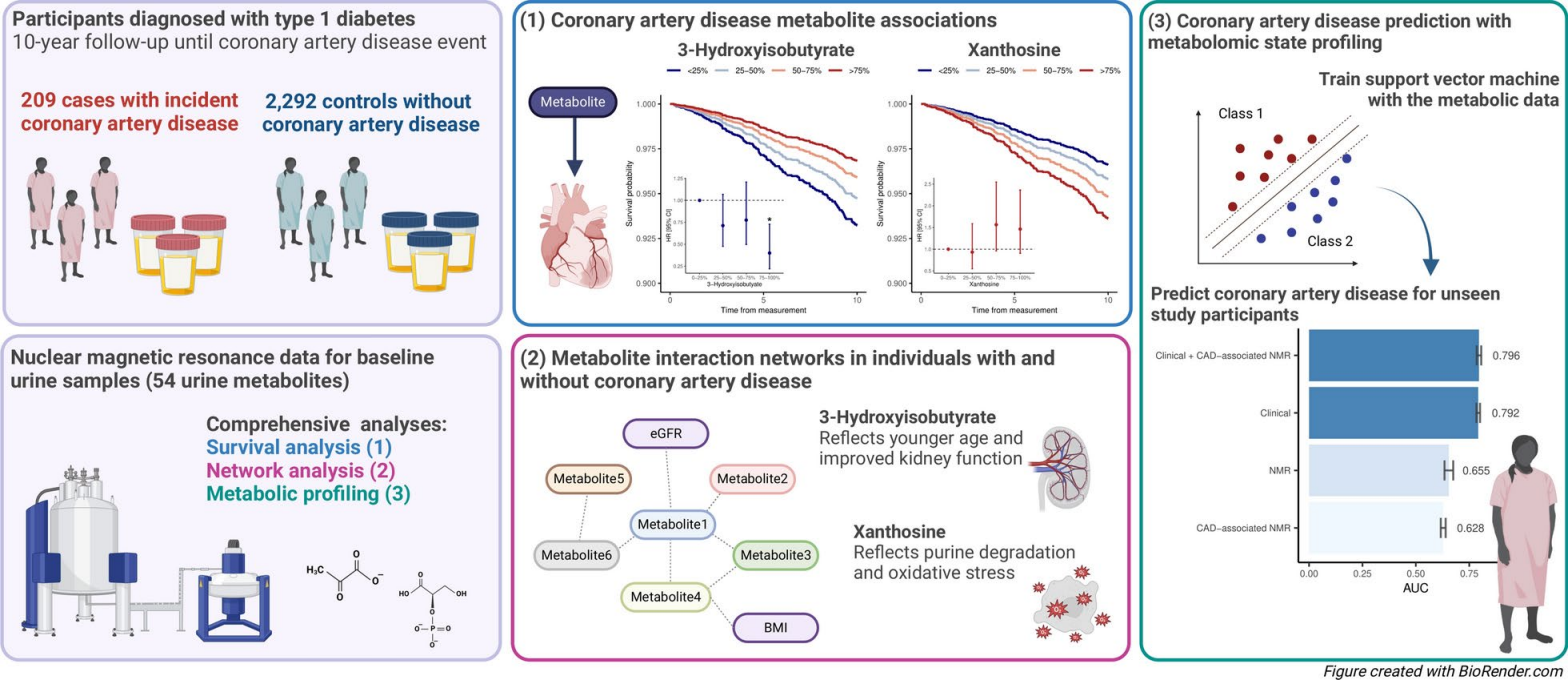

**Supplementary Information:**

The online version contains supplementary material available at 10.1186/s12933-024-02512-8.

## Background

Type 1 diabetes is characterized by the destruction of pancreatic *β*-cells and insulin deficiency [1]. Diabetes predisposes to cardiovascular disease (CVD), and despite careful blood glucose monitoring and insulin treatment, the risk of developing coronary artery disease (CAD) and other life-threatening complications remains [2, 3]; the life-time risk is particularly high in type 1 diabetes due to early age at diabetes onset [4]. Among individuals with type 1 diabetes, CAD is the leading cause of death [5]. The standardized incidence ratio for CAD has been estimated to be 4.0 for individuals with type 1 diabetes and normal kidney function in comparison to controls without diabetes, and the incidence ratio increases as the kidney function declines, reaching 26.6 at kidney replacement therapy (KRT) [6]. Many pathophysiological mechanisms may be involved, e.g., inflammation, hyperglycemia and oxidative stress, advanced glycation end-products (AGEs), dyslipidemia, decline in kidney function, and hypertension [7].

The metabolome provides a snapshot of the body’s physiological state [8]. Large-scale metabolomic studies aim to identify disease-associated metabolites within a hypothesis free framework, in hope that the discovered metabolites would provide insight into disease pathophysiology and serve as predictive markers in clinical practice. Here, we study urinary metabolomics measured by nuclear magnetic resonance (NMR) spectroscopy [9]. Urine is of interest due to its intrinsic properties: Metabolites are less strongly regulated within urine than in blood [10], and the weaker intercorrelations between urinary metabolites provide independent information [11]. The kidneys’ attempt to filter harmful or unnecessary substrates, and to eliminate them from the blood for as long as possible, may enable earlier disease detection than in blood [12].

Early identification of individuals at high risk of CAD would enable early targeting of modifiable risk factors (e.g., hypertension and dyslipidemia [7]) with medication and life-style, aiming at disease prevention. Clinical risk prediction models have been proposed for CVD prediction in type 1 diabetes [13, 14], and we have shown that genetic risk scores can further improve CAD prediction in type 1 diabetes [15]. Prediction with omics data may further improve prediction accuracy, e.g., serum metabolomic profiles perform well in disease prediction, including CAD [16].

In the general population, the metabolomics of CVD has been mainly studied in blood and heart tissue [17]. CVD has been associated with multiple plasma metabolites, e.g., pyrimidine metabolism and tricarboxylic acid cycle (TCA) metabolites, branched-chain amino acids, short-chain dicarboxylacylcarnitine, and trimethylamine N-oxide [17]. Urinary metabolomics have identified, e.g., important associations between formate and blood pressure [18]. However, only a few urinary metabolites have been associated– mainly cross-sectionally– with CAD in the general population, e.g., metabolites related to histidine and arginine–proline metabolism [19, 20]. Urinary metabolomics have not been studied for CAD in type 1 diabetes, but hold special promise due to the importance of kidney health in diabetes [6]. Finally, metabolomic state profiling using machine learning, i.e., integrating metabolomic states and evaluating their predictive accuracy, has been gaining attention in precision medicine [16, 21]. Here, we report the first large-scale urinary metabolomics analysis of CAD in diabetes, aiming to identify disease-associated metabolites and to perform metabolomic state profiling for CAD prediction.

## Methods

### Study cohort

**Fig. 1:**
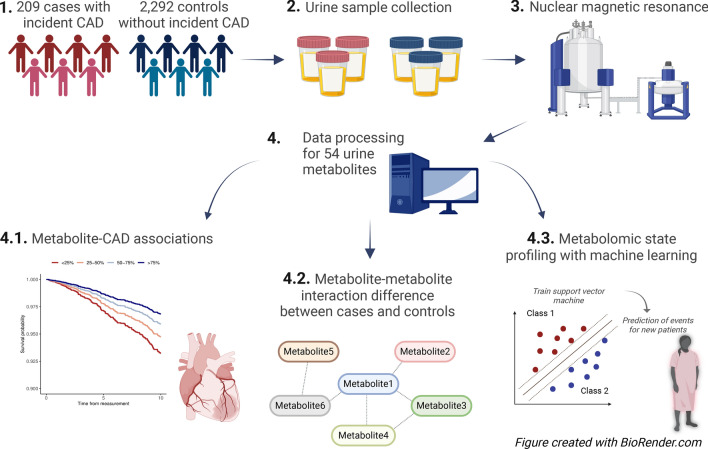
Study design with the 10-year incident coronary artery disease (CAD) cohort

The study includes 3111 adults from the Finnish Diabetic Nephropathy (FinnDiane) Study, an ongoing, nationwide study on the complications of type 1 diabetes; described in depth earlier [22]. The study was approved by the Ethics Committee of the Helsinki and Uusimaa Hospital District (491/E5/2006, 238/13/03/00/2015, HUS-3313-2018), and performed in accordance with the Declaration of Helsinki, with written informed consent obtained from the participants. Participants underwent a clinical examination and provided blood and urine samples at baseline. In this study, participants had been diagnosed with type 1 diabetes by their attending physician before the age of 40 years and with insulin treatment initiated within a year from diagnosis. At baseline, we excluded individuals with diabetes duration ≤ 0.5 years, CAD before baseline, or kidney failure (i.e., KRT or estimated glomerular filtration rate (eGFR) < 15) (Additional file 1: Fig. S1). CAD was defined as myocardial infarction, percutaneous coronary intervention, or coronary artery bypass graft, according to Finnish national registries (Additional file 1: Table S1). Cases were diagnosed by CAD during follow-up. Controls were not diagnosed by CAD of any severity (ICD-10: I20-I25) or heart failure during follow-up, and had age ≥ 35 years and diabetes duration ≥ 15 years at the end of the follow-up. In the main analysis, participants were followed until a CAD event, death, end of 2020, or reaching a maximum of 10 years follow-up. With this main cohort (N = 2501, follow-up: 9.1 ± 2.1 years, Additional file 1: Fig. S2-S3), we performed metabolome-wide survival analysis, metabolite-metabolite network analysis, and metabolomic state profiling (Fig. 1). Finally, we performed metabolome-wide survival analysis with the full follow-up, with partly different study subjects due to the follow-up dependent control inclusion criteria (N = 2953, follow-up: 17.0 ± 5.9 years, Additional file 1: Table S2). Ideally, the reported associations should be replicated in an external cohort. To our knowledge, similar urinary metabolomics panels are currently not measured in other diabetes cohorts that would allow diabetes-specific prospective replication on CAD, and therefore, the reported associations lack external replication.

### Nuclear magnetic resonance spectroscopy

Samples were analyzed with proton NMR at Nightingale Health, Helsinki, Finland, capturing 54 metabolites (mmol/l), as described in detail previously [10]. In the metabolite selection, Nightingale Health has favored metabolites with high abundance in the urine and with minimal signal overlap in the proton NMR spectrum [10, 11]; selection was not based on prior information about biological relevance [10, 11]. Most urine samples were collected as 24-h urine collections (56 overnight samples) and had been stored at −20 °C for on average 17 (± 4) years. We replaced metabolite zero concentrations with the metabolite’s half the minimum detected value, seen as the detection limit, and divided each metabolite concentration with the sample’s urinary creatinine concentration as a normalization procedure for urine flow rate (Additional file 1: Table S3). We transformed metabolite-to-creatinine ratios with logarithm or square root, as seen appropriate, and standardized to zero mean and unit variance (Additional file 1: Fig. S4).

### Metabolome-wide survival analysis

We performed metabolome-wide Cox proportional hazards survival modeling with R 4.3.0 (survival package 3.5–5) [23–25]. We selected the 10-year CAD risk modeling scheme due to a good trade-off between the case-control ratio and the proportion of metabolites fulfilling the proportional hazards assumption. Adjustment covariates fulfilled the proportional hazards assumption when inspected individually. The metabolome-wide survival analyses were adjusted, first, for non-modifiable risk factors (i.e., age, sex, diabetes onset calendar year); second, additionally for kidney function (i.e., eGFR, albuminuria), and finally, additionally to the previous for established CAD risk factors (i.e., glycated hemoglobin (HbA_1c_, %), low-density lipoprotein (LDL) cholesterol, waist-to-height ratio (WHtR), systolic blood pressure (SBP), and smoking) [13, 26]. For metabolites displaying non-proportional hazards within a specific adjustment setting, we additionally considered 5-year survival modeling without re-applying the follow-up dependent control inclusion criteria (N_case/control_ = 98/2403, follow-up: 4.8 ± 0.6 years). The 5-year taurine model was globally non-proportional, induced by HbA_1c_. Thus, we stratified HbA_1c_ in this model (< 6, 6–7, 7–8, 8–9, 9–10, 10–11, > 11, %).

We performed stratified analyses in individuals with (N_case/control_ = 106/673) and without albuminuria (N_case/control_ = 103/1619); defined as albumin excretion rate > 30 mg/24 h or equivalent in two out of three consecutive measurements (Additional file 1: Table S4), and assessed an interaction effect with albuminuria (N = 2501). We adjusted these models for the non-modifiable risk factors, kidney function, and established CAD risk factors; no adjustment covariate required stratification.

Metabolome-wide significance threshold was defined with the number of metabolites required to explain ~ 99% of variance within the metabolome according to principal component analysis (N = 46, Additional file 1: Fig. S5). Missing values were imputed to the mean. Taurine, creatine, and mannitol were excluded due to missingness rate > 50%. Thus, we incremented 46 by three, when defining the metabolome-wide significance threshold: 0.05/49 = 0.00102.

The full follow-up survival modeling was performed with 37 metabolites displaying proportional hazards in non-adjusted models, and a re-defined significance threshold (*p* < 0.05/37 = 0.00135). Age displayed non-proportional hazards; thus, we stratified it (< 30, 30–40, 40–50, 50–60, > 60 years). Mannitol model was globally non-proportional in the full adjustment setting, induced by WHtR, where we, thus, stratified it (< 0.45, 0.45–0.50, 0.50–0.55, 0.55–0.60, > 0.60).

As 3-hydroxyisobutyrate was previously associated with insulin resistance [27], we inspected its association with baseline insulin sensitivity using linear regression. We used the HbA_1c_-adapted formula from Williams et al. (2000) for estimated glucose disposal rate (eGDR) as a proxy for insulin sensitivity: 24.4–12.97 × waist-to-hip ratio–3.39 × AHT–0.60 × HbA_1c_ (%), where AHT is antihypertensive treatment and/or blood pressure ≥ 140/90 mmHg (yes = 1, no = 0) [22, 28].

### Metabolite-metabolite network

We describe metabolite interactions with networks including the baseline clinical variables, i.e., sex, age, diabetes duration, diabetes onset calendar year, diabetes onset age, albuminuria (yes/no), eGFR, HbA_1c_ (%), SBP, diastolic blood pressure (DBP), mean arterial pressure (MAP), triglyceride (log-transformed), total cholesterol, LDL cholesterol, high-density lipoprotein (HDL) cholesterol, body-mass-index (BMI), WHtR, and smoking (current or history, yes/no). Here, network is a representation of a correlation coefficient matrix, i.e., variables are the network nodes, and the correlation coefficients between them are the weighted network links. We utilized Spearman’s rank correlation for continuous-continuous pairings, point-biserial correlation for dichotomous-continuous pairings, and phi coefficient for dichotomous-dichotomous pairings, by excluding missing data. We calculated three baseline correlation matrices with the 10-year follow-up cohort: complete cohort, incident CAD cases, and controls. Furthermore, we evaluated correlation difference between cases and controls by defining significance with random permutations (B = 20000), resulting in the precision limit of *p* > 5 × 10^–5^ (1/20001) [29]. Baseline correlation networks include significant links (*p* < 2 × 10^–5^, 72 nodes, 2556 possible links). Of note, statistical power to detect a metabolite-metabolite correlation with the 2 × 10^–5^ significance threshold is approximately 80% for correlations of magnitude 0.34, 0.11, and 0.10 in case-, control- and complete baseline networks, respectively. The case-to-control correlation difference network includes nominally significant links (*p* < 0.05), whenever correlation was significant in cases and/or controls (*p* < 2 × 10^–5^). We evaluated node importance with degree centrality (number of connections) and betweenness centrality (how often the node lies within a shortest path connecting two other nodes) and described general network connectedness with global clustering coefficient.

### Metabolomic state profiling

We trained support vector machine (SVM) models, aiming to predict 10-year and 5-year incident CAD, with cohorts defined correspondingly to the survival analysis. SVM can capture non-linearity unlike logistic regression and functions better with a high-dimensional and/or noisy data [30]. Therefore, we estimated how beneficial the urinary metabolites could be on top of the clinical risk factors, with a more complex mathematical approach than the logistic regression, for future studies that aim to build validated clinical risk scores. We excluded variables with missingness rate > 15% (allantoin, 3-aminoisobutyrate, creatine, ethanolamine, ethanol, glycolic acid, histidine, hypoxanthine, isoleucine, mannitol, propylene glycol, proline betaine, taurine, 3-methylhistidine, xylose, smoking, and WHtR), and imputed the remaining missing data to the variable mean. We trained four models: 1. CAD-associated NMR (metabolites associated with incident CAD after full adjustment, *p* < 0.05), 2. NMR (all urinary metabolites), 3. Clinical variables (clinical variables corresponding to network analysis), 4. Clinical variables + CAD-associated NMR.

We performed training and validation within ten bootstraps (i.e., randomly selected sub-samples). For the 10-year CAD SVM, 208 participants were sampled randomly without replacement into training (N_case/control_ = 104/104), and the remaining were assigned into independent validation (N_case/control_ = 105/2188, Additional file 1: Fig. S6). For the 5-year CAD SVM, we similarly sampled 98 participants into training (N_case/control_ = 49/49) and assigned the remaining into validation (N_case/control_ = 49/2354). We utilized non-linear radial basis kernel with variable scaling and C-classification. Thus, we optimized the kernel’s γ parameter [1, 0.5, 0.1, 0.05, 0.01, 0.005, 0.001] and SVM’s C constant [1, 5, 10, 15, 20, 25, 30, 35, 40, 45, 50] with grid search*.* Within the training data, 50% of participants were randomly sampled without replacement to *pre-training*, to train SVMs with different parameters, and the rest were assigned into *pre-training* validation, where we selected the best model parameters using phi coefficient. To improve variable selection stability, we performed *pre-training* within ten bootstraps, and considered the median parameter specific performance in grid search. We then performed SVM modeling with bagging: 100 SVMs were trained with the selected parameters and with 100% of the training data randomly sampled with replacement. In the independent validation in each bootstrap, we predicted incident CAD with the 100 SVMs and considered the percentage of SVMs supporting incident CAD as the prediction score (i.e., ensemble learning).

## Results

### Metabolome-wide survival analysis

**Fig. 2:**
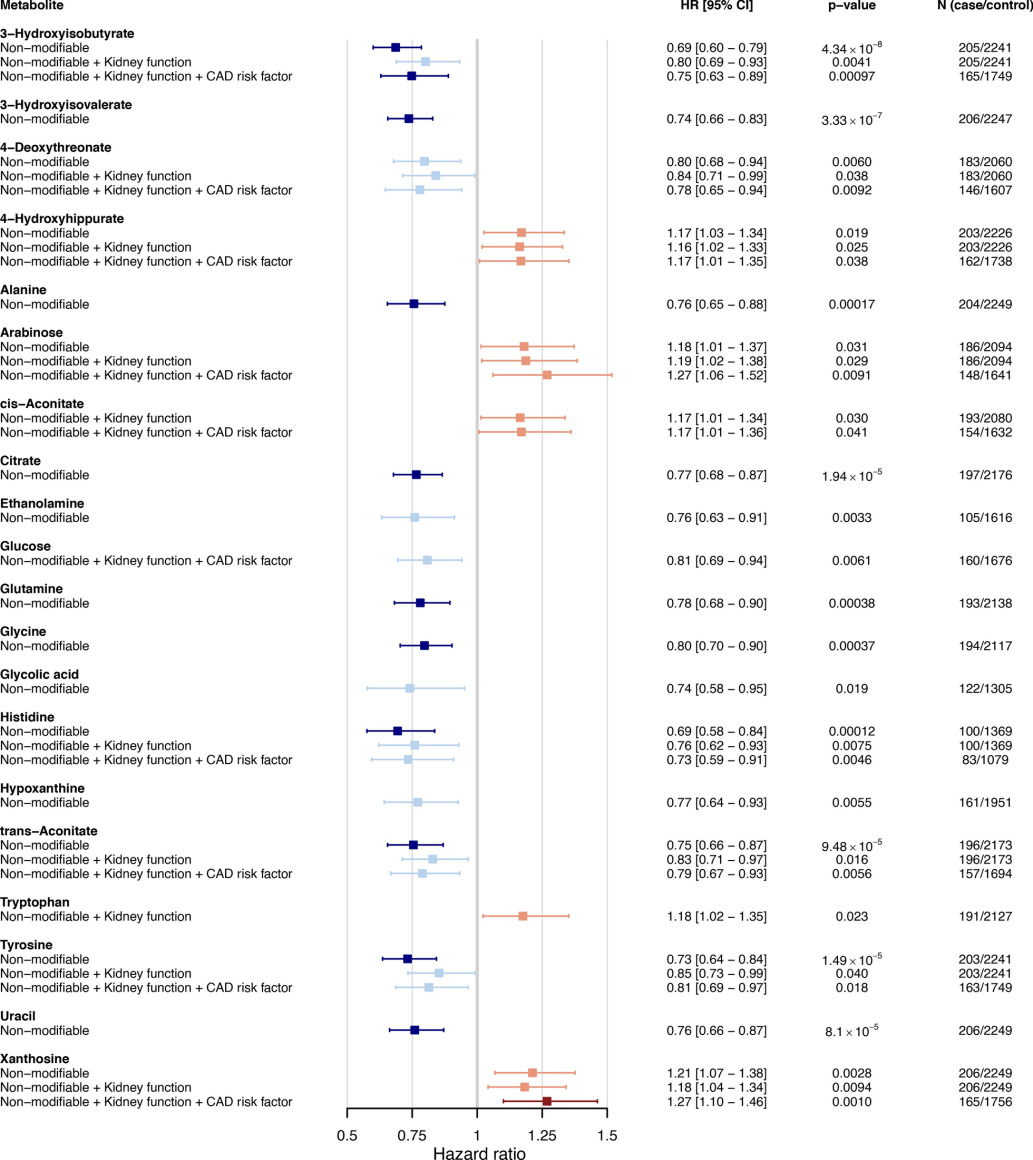
Metabolome-wide survival analysis for 10-year coronary artery disease (CAD) risk in individuals with type 1 diabetes (N = 2501). Survival analyses with adjustment settings: **1.** non-modifiable risk factors (age, sex, diabetes onset calendar year), **2.** non-modifiable risk factors + kidney function (eGFR, albuminuria), **3.** non-modifiable risk factors + kidney function + CAD risk factors (systolic blood pressure, LDL cholesterol, waist-to-height ratio, HbA_1c_, smoking). Forest plot displays metabolite-CAD associations (*p* < 0.05). Darker colors indicate metabolome-wide significant associations (*p* < 0.00102)

**Fig. 3:**
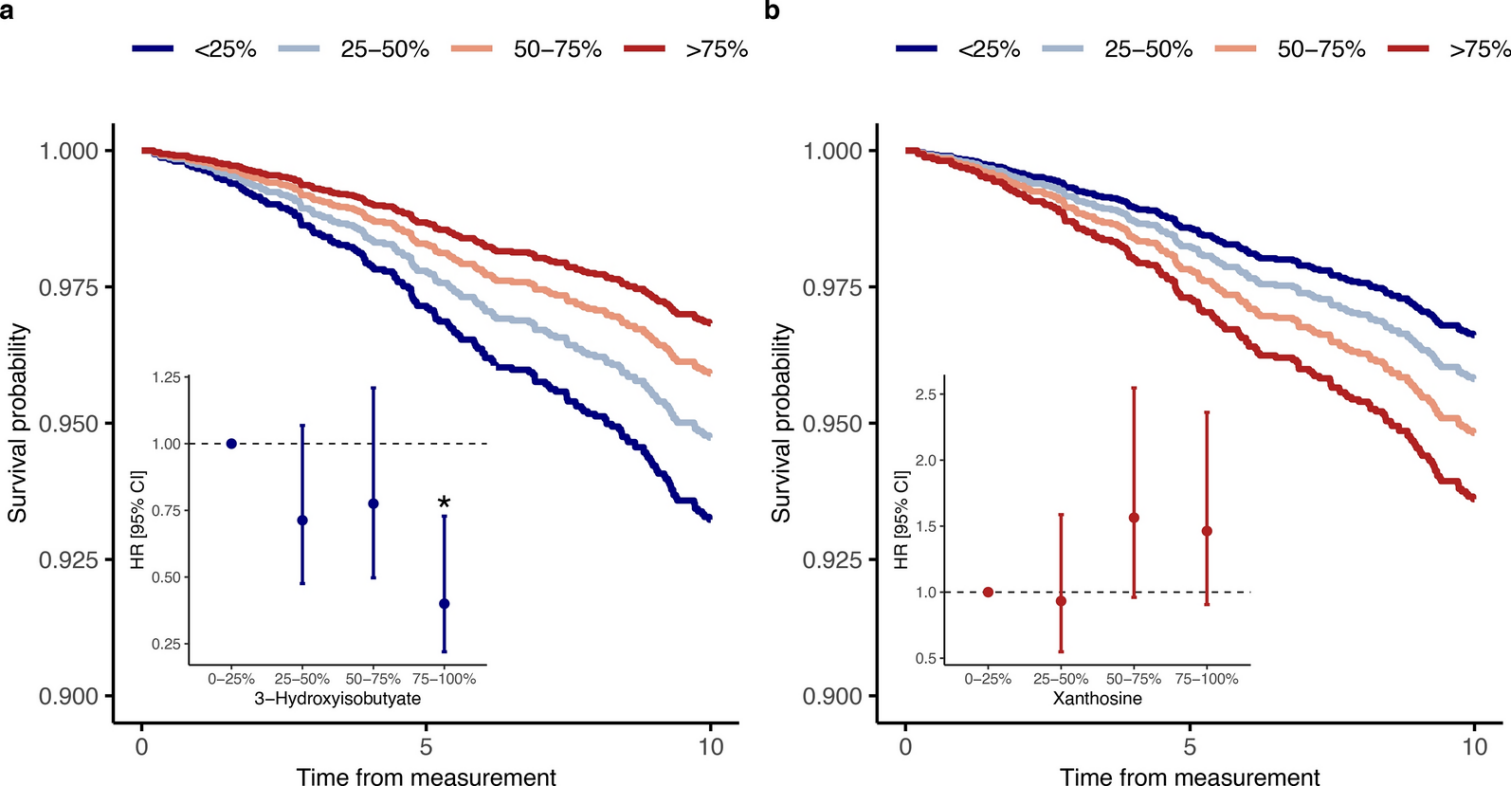
3-hydroxyisobutyrate (**a**) and xanthosine (**b**) quartile’s survival probability within the 10-year CAD risk Cox proportional hazard survival models adjusted for age, sex, diabetes onset calendar year, eGFR, albuminuria, HbA_1c_, systolic blood pressure, LDL cholesterol, waist-to-height ratio, and smoking. Inserted are the hazard ratios for 10-year incident CAD, correspondingly adjusted, between individuals belonging to the higher metabolite quartiles (25–50%, 50–75%, 75–100%) and individuals belonging to the lowest quartile (0–25%); *statistically significant (*p* < 0.05)

The 10-year survival modeling included 2501 individuals with type 1 diabetes of whom 209 experienced incident CAD (Table 1, Additional file 1: Fig. S2-S3). Ten urinary metabolites were metabolome-wide significantly associated with incident CAD in type 1 diabetes after adjustment for the non-modifiable risk factors (*p* < 0.00102): 3-hydroxyisobutyrate, 3-hydroxyisovalerate, alanine, citrate, glutamine, glycine, histidine, trans-aconitate, tyrosine, and uracil (Fig. 2). In addition, seven metabolites were nominally associated with incident CAD in type 1 diabetes (*p* < 0.05). No metabolites remained metabolome-wide significant after additional adjustment for kidney function, however, ten metabolites showed nominal association (*p* < 0.05). With the survival models adjusted for the non-modifiable risk factors, kidney function, and the established CAD risk factors, we found 3-hydroxyisobutyrate (HR = 0.75 [0.63–0.89] per 1SD, *p* = 9.7 × 10^–4^) and xanthosine (HR = 1.27 [1.10–1.46] per 1SD, *p* = 0.0010) to associate metabolome-wide significantly with incident CAD in type 1 diabetes, and eight metabolites to be nominally associated (*p* < 0.05) (Fig. 3). A stronger control of phenotypic dispersion with more comprehensive adjustment could explain the emergence of associations missed with less stringent adjustment.

**Table 1:** Baseline clinical characteristics for cases and controls within the 10-year follow-up (mean ± SD)

	Cases with incident CAD	Controls without incident CAD	*p*-value	Missing (N)
N	209	2292		
Sex (male, %)	110 (53%)	1115 (49%)	0.30	
Diabetes onset calendar year*	1967 ± 13	1978 ± 15	6.54 × 10^–36^	
Age (year)*	50.36 ± 13.65	39.22 ± 14.63	3.02 × 10^–33^	
Diabetes duration (year)*	33.38 ± 11.79	22.59 ± 14.45	1.92 × 10^–32^	
Age at diabetes onset (year)*	15.29 ± 13.80	15.54 ± 13.54	1.00	
Estimated glomerular filtration rate (ml/min/1.73 m^2^)*	75 ± 41	94 ± 31	4.91 × 10^–20^	
HbA_1c_ (mmol/mol)	71.31 ± 15.90	66.67 ± 14.26	8.04 × 10^–5^	127
HbA_1c_ (%)	8.67 ± 1.45	8.25 ± 1.30		
Systolic blood pressure (mmHg)	145 ± 19	133 ± 17	2.13 × 10^–15^	262
Diastolic blood pressure (mmHg)	79 ± 11	80 ± 9	0.39	262
Mean arterial pressure (mmHg)	101 ± 11	98 ± 10	2.33 × 10^–5^	262
Total cholesterol (mmol/l)	5.30 ± 1.08	4.92 ± 0.88	1.47 × 10^–6^	6
HDL cholesterol (mmol/l)	1.30 ± 0.39	1.40 ± 0.40	0.00026	7
Triglyceride (mmol/l)*	1.20 ± 0.69	0.94 ± 0.60	1.88 × 10^–12^	6
LDL cholesterol (mmol/l)**	3.40 ± 0.92	3.03 ± 0.82	9.65 × 10^–8^	7
Body-mass-index (kg/m^2^)	25.70 ± 3.64	25.54 ± 3.61	0.56	278
Waist-to-height ratio	0.53 ± 0.07	0.51 ± 0.06	1.00 × 10^–5^	400
Smoking (current or history, %)	82 (46%)	874 (45%)	0.98	397
Antihypertensive medication (yes, %)	123 (68%)	705 (36%)	7.17 × 10^–17^	351
Lipid-lowering medication (yes, %)	52 (29%)	203 (10%)	7.40 × 10^–13^	350
Albuminuria (yes, %)	106 (51%)	673 (29%)	2.90 × 10^–10^	

*Median ± IQR, ^**^LDL cholesterol calculated with Sampson formula

Furthermore, we noticed a negative relationship between the metabolites’ correlation with eGFR and the hazard ratios for CAD (Additional file 1: Fig. S7), which was especially strong before adjusting the hazard ratios for kidney function (*r* = −0.74, *p* = 1.31 × 10^–10^). This trend is particularly detectable with the CAD-protective metabolites. Although the relationship weakens along with kidney function adjustment, a more subtle relationship may still be observed after full adjustment (*r* = −0.52, *p* = 5.78 × 10^–5^). Therefore, CAD-protective metabolite association could be slightly overestimated.

Serum 3-hydroxyisobutyrate has been shown to associate with insulin resistance [27], but our data contradictorily proposed lower urinary 3-hydroxyisobutyrate to associate with incident CAD in type 1 diabetes. Therefore, we evaluated the urinary 3-hydroxyisobutyrate association with the baseline eGDR, which is used to estimate insulin sensitivity in individuals with type 1 diabetes [22, 28]. 3-Hydroxyisobutyrate was associated with lower eGDR, i.e., higher insulin resistance, after adjustment for non-modifiable risk factors and kidney function (β = −0.19 per 1SD, *p* = 1.12 × 10^–5^, Additional file 1: Table S5), in line with the previous research. Thus, 3-hydroxyisobutyrate likely conveys it’s CAD-protective effect through a protective pathway, which is independent of insulin resistance.

Seven metabolites displayed non-proportional hazards within at least one of the adjustment settings (Additional file 1: Fig. S8). We additionally considered 5-year survival modeling for these seven metabolites, and found decreased 4-deoxythreonate (HR = 0.60 [0.45–0.79] per 1SD, *p* = 0.00032) and trans-aconitate (HR = 0.66 [0.53–0.82] per 1SD, *p* = 0.00014) to associate with 5-year incident CAD in type 1 diabetes metabolome-wide significantly after adjustment for the non-modifiable risk factors, kidney function, and the established CAD risk factors (*p* < 0.00102, Additional file 1: Table S6).

Furthermore, we performed survival modeling with the full follow-up up to 23 years (17.0 ± 5.9 years, N_case/control_ = 496/2457, Additional file 1: Fig. S9-S10). We found arabinose, dimethylamine, and uracil to be nominally associated with incident CAD in type 1 diabetes after adjustment for the non-modifiable risk factors, kidney function, and the established CAD risk factors (*p* < 0.05).

**Fig. 4:**
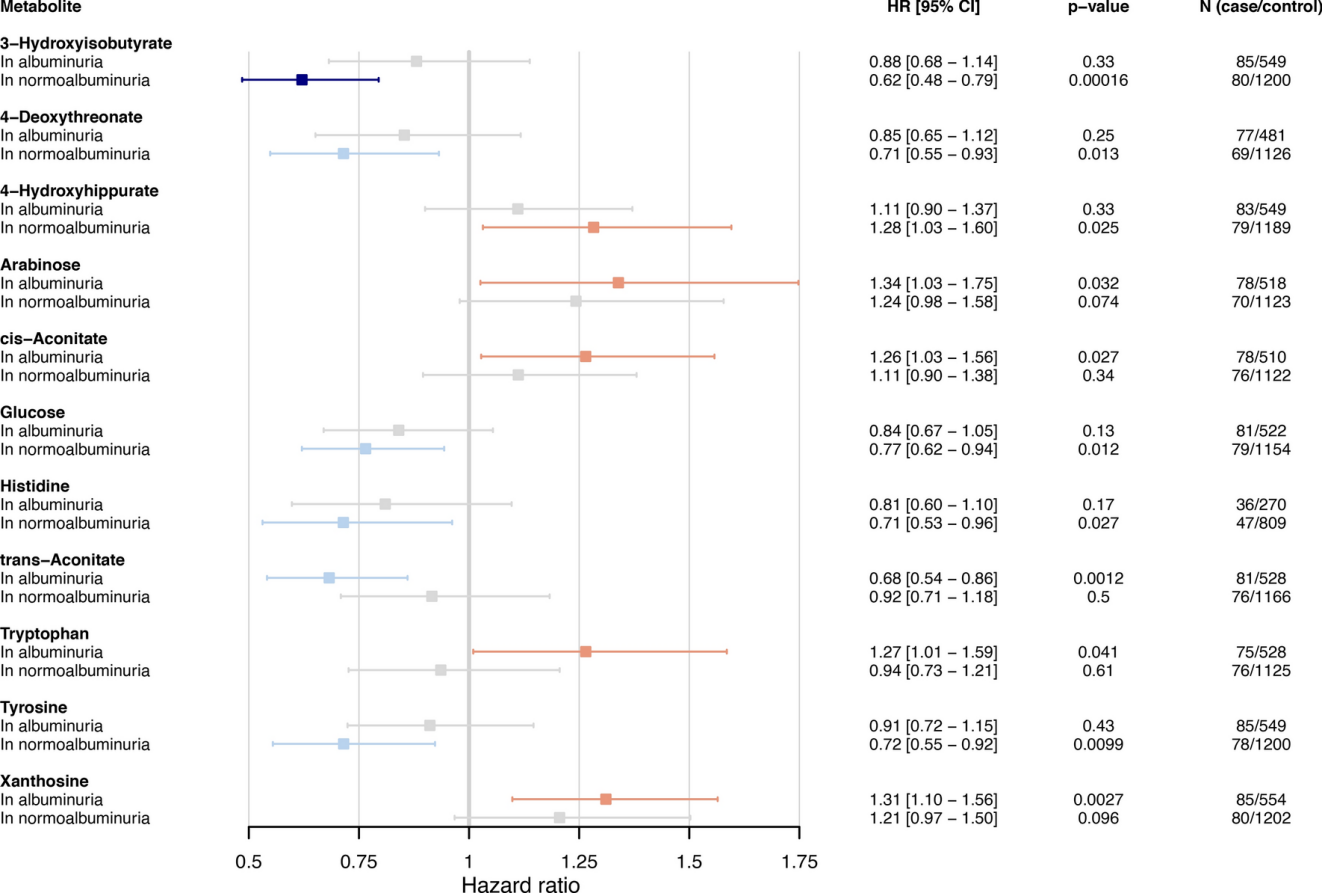
Metabolome-wide survival analysis for 10-year coronary artery disease (CAD) risk in individuals with type 1 diabetes and stratified into participants with and without albuminuria (i.e., albuminuria vs. normoalbuminuria). Adjusted for age, sex, diabetes onset calendar year, eGFR, systolic blood pressure, LDL cholesterol, waist-to-height ratio, HbA_1c_, smoking. Forest plot displays metabolite-CAD associations, whenever significant in individuals with or without albuminuria (*p* < 0.05). Darker color indicates metabolome-wide significance (*p* < 0.00102). Metabolite-albuminuria interaction effects on 10-year CAD were non-significant (*p* > 0.05)

As diabetic kidney disease (DKD) is a major CAD risk factor in diabetes [6], we additionally performed 10-year survival modeling stratified by albuminuria status in type 1 diabetes. In the full adjustment model, decreased 3-hydroxyisobutyrate associated metabolome-wide significantly with CAD in individuals without albuminuria (HR = 0.62 [0.48–0.79] per 1SD, *p* = 0.00016), but not in individuals with albuminuria (Fig. 4). On the contrary, xanthosine was nominally associated with CAD in individuals with albuminuria (HR = 1.31 [1.10–1.56] per 1SD, *p* = 0.0027), but not in individuals without albuminuria. However, we did not detect significant metabolite × albuminuria interaction effects on incident CAD. Eight metabolites displayed non-proportional hazards. We considered 5-year survival modeling when needed and found decreased trans-aconitate to associate significantly with CAD in individuals with albuminuria (HR = 0.53 [0.39–0.71] per 1SD, *p* = 3.09 × 10^–5^, Additional file 1: Table S7), and to display an interaction effect with albuminuria on 5-year incident CAD (*p* < 0.05).

### Metabolite-metabolite network

**Fig. 5:**
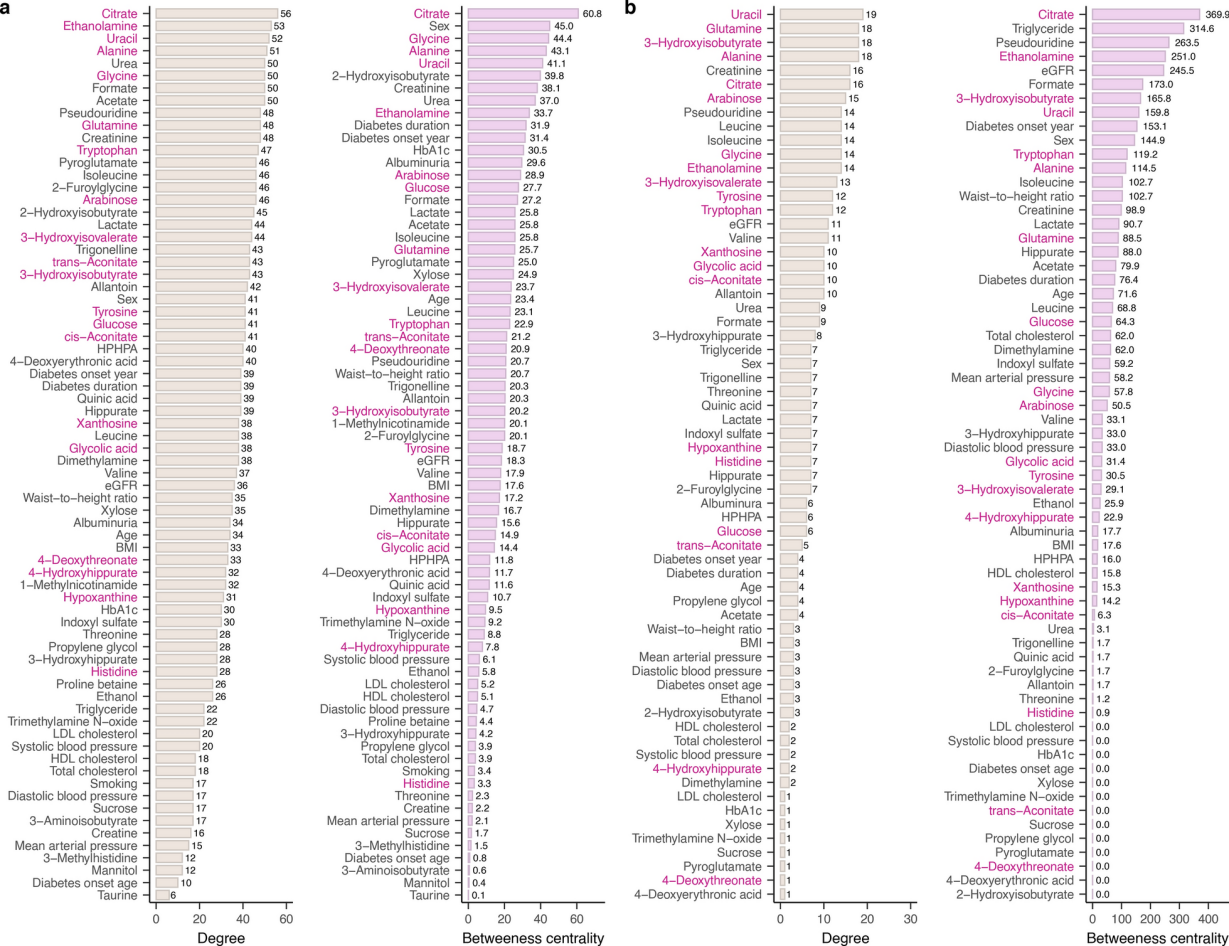
Local centrality measures for the complete baseline metabolic correlation network (*p* < 2 × 10^–5^, N_patient_ = 2501) (**a**), and the cases’ baseline metabolic correlation network (*p* < 2 × 10^–5^, N_patient_ = 209) (**b**). Here, degree is the number of links leaving a node. Betweenness centrality describes the amount of non-weighted network shortest paths between other nodes passing through the node. 10-year coronary artery disease risk associated metabolites are highlighted with pink (*p* < 0.05). HPHPA: 3-(3-hydroxyphenyl)-3-hydroxypropionic acid

To describe metabolite interactions with other metabolites and clinical characteristics, we performed a correlation network analysis with the 10-year follow-up metabolomic data in type 1 diabetes (N = 2501, Additional file 1: Fig. S11, Additional file 2). Thus, network is a visualization of the correlation matrix, and can help to highlight mechanistically interesting links between metabolites and clinical variables (“nodes”), while using “centrality” (i.e., connectedness with other nodes) to describe variable importance. We found the urinary metabolome to be highly interconnected (global clustering coefficient = 0.64, Additional file 1: Table S8). Sex was the most central clinical variable (Fig. 5). The most central metabolites were citrate, ethanolamine, uracil, alanine, urea, and glycine (degree centrality ≥ 50); all except urea were associated with incident CAD in survival analysis after non-modifiable risk factor adjustment.

3-Hydroxyisobutyrate and xanthosine, which associated with 10-year incident CAD in the fully adjusted survival models, displayed high centrality. 3-Hydroxyisobutyrate correlated with 37 other metabolites (*p* < 2 × 10^–5^), the most strongly with 3-hydroxyisovalerate (*r* = 0.62). Out of the clinical variables, 3-hydroxyisobutyrate correlated positively with diabetes onset calendar year (*r* = 0.15), eGFR (*r* = 0.26), and HbA_1c_ (*r* = 0.11), and negatively with age (*r* = −0.14), diabetes duration (*r* = −0.19), and albuminuria (*r* = −0.25). Xanthosine correlated with 31 other metabolites (*p* < 2 × 10^–5^), the strongest with arabinose (*r* = 0.68). Out of the clinical variables, xanthosine correlated positively with female sex (*r* = 0.24), age (*r* = 0.096), diabetes duration (*r* = 0.16), and HDL cholesterol (*r* = 0.11), and negatively with diabetes onset calendar year (*r* = −0.095), HbA_1c_ (*r* = −0.089), and BMI (*r* = −0.11).

As the metabolites were normalized by urinary creatinine concentrations, the clinical correlations may partly reflect inverse correlation to urine creatinine, especially with creatinine-associated variables, e.g., age and BMI [31, 32]. Thus, as a sensitivity analysis for the clinical variable associations of xanthosine and 3-hydroxyisobutyrate, we reconsidered them without the creatinine normalization in regression with urinary creatinine as independent variable, as proposed previously [31] (Additional file 1: Table S9). We found xanthosine to correspondingly associate with female sex, longer diabetes duration, and improved HbA_1c_ (*p* < 2 × 10^–5^), but not with the other clinical variables (*p* > 2 × 10^–5^). 3-Hydroxyisobutyrate displayed associations in correspondence to the network (*p* < 2 × 10^–5^), except being additionally negatively associated with SBP (*p* < 2 × 10^–5^).

Importantly, neither trans-aconitate nor 4-deoxythreonate correlated significantly with eGFR in the network (*p* > 2 × 10^–5^). trans-Aconitate correlated with 40 other metabolites (*p* < 2 × 10^–5^), the strongest with ethanolamine (*r* = 0.38). 4-Deoxythreonate correlated with 28 other metabolites (*p* < 2 × 10^–5^), the strongest with 2-hydroxyisobutyrate (*r* = 0.41).

Furthermore, we evaluated a correlation network including only individuals with 10-year incident CAD (Additional file 1: Fig. S12). With smaller sample size (N = 209), we observed fewer significant correlation coefficients (*p* < 2 × 10^–5^), and the network was no longer as interconnected (global clustering coefficient = 0.54, Additional file 1: Table S8). Triglycerides and eGFR were highly central (Fig. 5). eGFR correlated significantly with eight metabolites (*p* < 2 × 10^–5^), including 3-hydroxyisobutyrate (*r* = 0.51), but not xanthosine, trans-aconitate or 4-deoxythreonate. 3-Hydroxyisobutyrate still correlated the strongest with 3-hydroxyisovalerate (*r* = 0.64). However, xanthosine now correlated the strongest with allantoin (*r* = 0.67), and trans-aconitate with citrate (*r* = 0.49). 4-Deoxythreonate correlated significantly only with 3-hydroxyisobutyrate (*r* = 0.37).

**Fig. 6:**
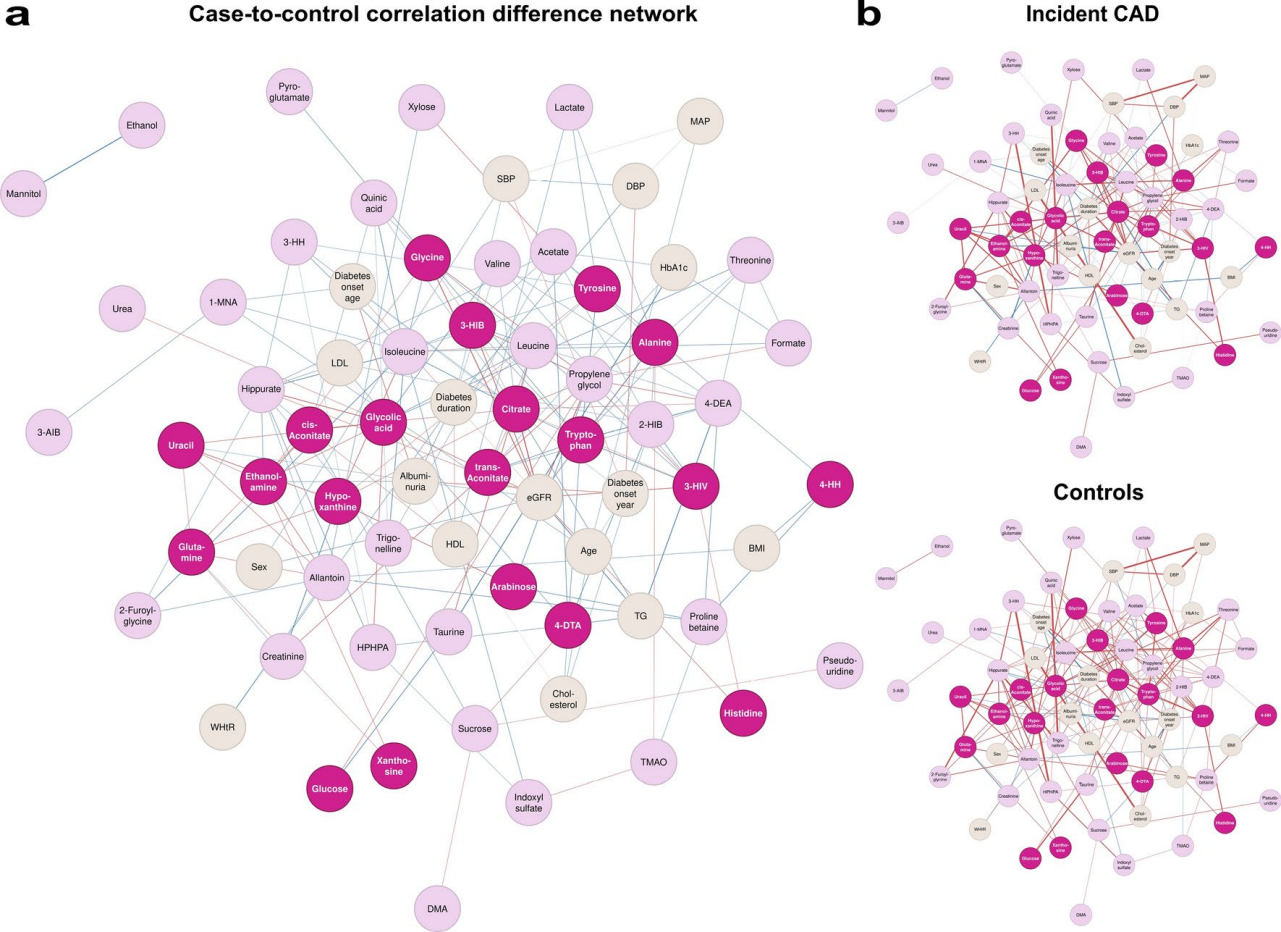
Correlation coefficient difference network between individuals with and without 10-year incident coronary artery disease (CAD) (*r*_case-to-control_, *p*_difference_ < 0.05, *p*_case or control_ < 2 × 10^–5^) (**a**). Red link represents a more positive correlation in individuals with than in individuals without incident CAD, blue link contrarily, and the link weight represent the magnitude. In addition, links included in the difference network are represented separately for individuals with (**b**) and without incident CAD (**c**); such that a red link represents a positive correlation coefficient, and a blue link negative. Clinical variable nodes are colored as beige and metabolite nodes as pink, darker pink for metabolites associated with 10-year incident CAD (*p* < 0.05). 4-Hydroxyhippurate (4-HH), dimethylamine (DMA), trimethylamine N-oxide (TMAO), 2-hydroxyisobutyrate (2-HIB), 3-hydroxyhippurate (3-HH), 3-(3-hydroxyphenyl)-3-hydroxypropionic acid (HPHPA), 3-aminoisobutyrate (3-AIB), 1-methylnicotinamide (1-MNA), 4-deoxyerythronic acid (4-DEA), 3-methylhistidine (3-MH), 4-deoxythreonate (4-DTA), 3-hydroxyisovalerate (3-HIV), 3-hydroxyisobutyrate (3-HIB), waist-to-height ratio (WHtR), triglyceride (TG), systolic blood pressure (SBP), diastolic blood pressure (DBP), mean arterial pressure (MAP)

We characterize correlation differences in type 1 diabetes between those with and without 10-year incident CAD with a case-to-control network visualization (Fig. 6, Additional file 1: Fig. S13-S14). We identified highly significant correlation differences between incident CAD cases and controls (*p* < 5 × 10^–5^); eGFR correlated more strongly with 3-hydroxyisobutyrate (*r*_case-to-control_ = 0.29), citrate (*r*_case-to-control_ = 0.42), and glycine (*r*_case-to-control_ = 0.35), while 4-deoxyerythronic acid correlated less strongly with triglycerides (*r*_case-to-control_ = −0.35), and hypoxanthine with creatinine (*r*_case-to-control_ = −0.30) in individuals with incident CAD. However, we considered also nominally significant differences in the case-to-control network (*p* < 0.05), and found citrate, isoleucine, leucine, hippurate and eGFR to be highly central (degree centrality ≥ 12). Interestingly, 3-hydroxyisobutyrate correlated less strongly with isoleucine and leucine in individuals with 10-year incident CAD (*p* < 0.05), but not with valine (*p* > 0.05). Of note, xanthosine correlated more strongly with glutamine, and trans-aconitate with citrate, in individuals with 10-year incident CAD. Finally, we highlight that 4-deoxythreonate correlated differently with serum triglycerides, cholesterol, and LDL cholesterol.

### Metabolomic state profiling

**Fig. 7:**
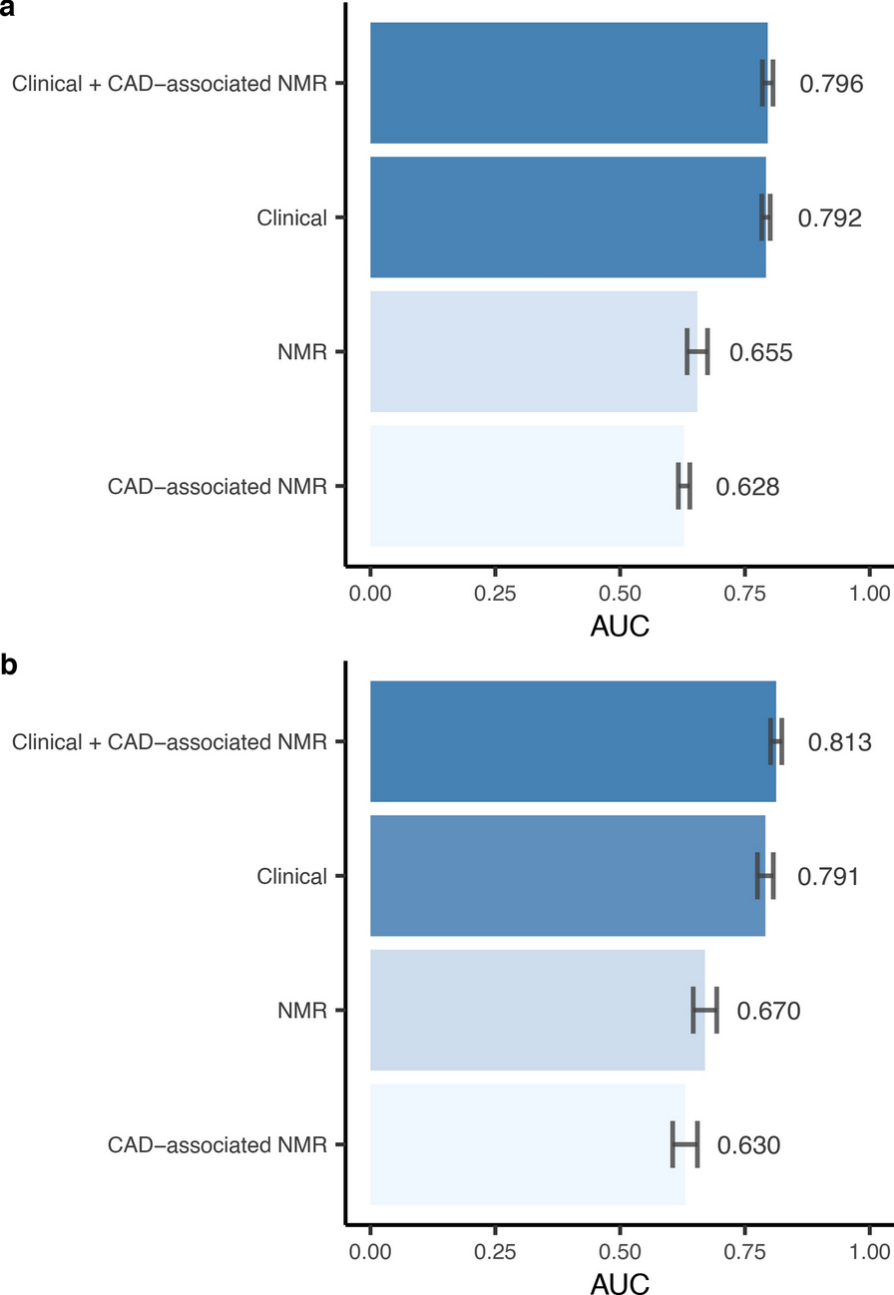
Coronary artery disease (CAD) prediction with support vector machine for 10-year incident CAD (**a**), and for 5-year incident CAD (**b**). Mean (± SD) area under receiver operating characteristic curve (AUC) across 10 bootstraps (i.e., replicate). **CAD-associated NMR**: Metabolites with missingness rate < 15% and associated with incident CAD after full adjustment (*p* < 0.05) (**a/b:** 3-hydroxyisobutyrate, xanthosine, 4-deoxythreonate, trans-aconitate, 4-hydroxyhippurate, arabinose, cis-aconitate, glucose, tyrosine, **b:** leucine). **NMR**: Metabolites with missingness rate < 15%. **Clinical**: sex, diabetes onset calendar year, diabetes onset age, age, diabetes duration, body-mass-index, systolic blood pressure, diastolic blood pressure, mean arterial pressure, triglyceride, total cholesterol, HDL cholesterol, LDL cholesterol, HbA_1c_, eGFR, albuminuria

We assessed the predictive ability of the urinary metabolome, at first for 10-year incident CAD in type 1 diabetes (Fig. 7a, Additional file 1: Fig. S15). SVM modeling with the nine CAD-associated metabolites performed reasonably well (AUC_N=10_ = 0.628). However, the model was outperformed by SVM modeling with all urinary metabolites (AUC_N=10_ = 0.655, *p* = 0.044). SVM modeling with the clinical variables predicted 10-year incident CAD in type 1 diabetes with a higher accuracy (AUC_N=10_ = 0.792). Cederholm et al. (2011) has proposed a clinical risk score for 5-year cardiovascular disease in type 1 diabetes [14]. We calculated it for the study participants and found it to predict 10-year incident CAD comparably to our clinical SVM model (AUC_N=10_ = 0.798, *p* = 0.36). Finally, SVM modeling with clinical variables and the CAD-associated metabolites modestly outperformed the clinical SVM model (AUC_N=10_ = 0.796), however, this improvement was not statistically significant (*p* = 0.63).

Urinary metabolites displayed stronger predictive ability for 5-year incident CAD in type 1 diabetes (Fig. 7b, Additional file 1: Fig. S16). SVM modeling with the ten CAD-associated metabolites (i.e., associated time-invariantly with 10-year or 5-year incident CAD), performed comparably to the 10-year predictive modeling scheme (AUC_N=10_ = 0.630). However, in the 5-year setting, the model was more clearly outperformed by modeling with all urinary metabolites (AUC_N=10_ = 0.670, *p* = 0.034). SVM modeling with the clinical variables performed comparably to the 10-year modeling scheme (AUC_N=10_ = 0.791), and again, with no significant difference to the Cederholm et al. (2011) clinical risk score (AUC_N=10_ = 0.794, *p* = 0.68). Importantly, modeling with the clinical variables and the CAD-associated metabolites did provide a significant improvement beyond the clinical SVM model (AUC_N=10_ = 0.813, *p* = 0.044).

## Discussion

We studied 54 urinary metabolites in relation to incident CAD in type 1 diabetes and found decreased 3-hydroxyisobutyrate and increased xanthosine to associate with 10-year incident CAD, and decreased trans-aconitate and decreased 4-deoxythreonate to associate with 5-year incident CAD, beyond the main clinical cardiovascular and kidney risk factors. Network analysis revealed metabolic and clinical connections, while metabolomic state profiling demonstrated that urinary metabolites can predict CAD and improve the prediction of 5-year CAD beyond the clinical risk factors.

3-Hydroxyisobutyrate– a valine degradation product– was associated with decreased risk of 10-year incident CAD in type 1 diabetes, especially among individuals without albuminuria. Contradictorily, valine is a well-established marker of insulin resistance [33], which has been associated with the progression of DKD in type 1 diabetes [10]. Serum 3-hydroxyisobutyrate has been associated with insulin resistance and type 2 diabetes [27, 34], and we confirmed that urinary 3-hydroxyisobutyrate was similarly associated with baseline insulin resistance in our data. Within the complete network, 3-hydroxyisobutyrate correlated positively with eGFR and HbA_1c_, and negatively with age; suggesting that urinary 3-hydroxyisobutyrate simultaneously reflects impaired glucose control, better kidney function, and younger age. Of note, serum 3-hydroxyisobutyrate has also previously been found to correlate negatively with age [35]. Glomerular filtration rate is an important predictor of CAD manifestations [36]. We hypothesize that higher glomerular filtration rate could increase particularly the small molecule excretion (e.g., 3-hydroxyisobutyrate, 103.10 g/mol), thus, also induce their higher urinary concentration. Within the case-to-control network comparison, 3-hydroxyisobutyrate linked to eGFR, but importantly, not to valine or HbA_1c_. Therefore, we hypothesize that the 3-hydroxyisobutyrate association with CAD reported here might reflect pathways related to younger age and/or better glomerular filtration rather than to metabolic syndrome, but we note that the association with CAD was significant independent of these baseline cardiovascular risk factors, suggesting also eGFR and age independent pathways. Therefore, further research is needed to characterize the putatively protective role of urinary 3-hydroxyisobutyrate on CAD in type 1 diabetes.

Xanthosine– a nucleoside composed of xanthine and ribose– was associated with increased risk of 10-year incident CAD in type 1 diabetes, specifically among individuals with albuminuria. We hypothesize that this observed xanthosine association reflects the purine degradation pathway: More specifically, the metabolism of guanosine monophosphate (GMP) and inosine monophosphate (IMP) to uric acid. IMP is transformed to hypoxanthine, which is further catabolized to uric acid through xanthine by a xanthine oxidoreductase (XOR) [37], which simultaneously generates reactive oxygen species [38]. GMP is transformed to guanine, which is also further metabolized to uric acid through xanthine partly by a XOR [37]. Importantly, the reactive oxygen species produced by XOR may lead to oxidative stress and to endothelial dysfunction [39, 40]. The downstream product, uric acid, is commonly elevated in serum of individuals with metabolic syndrome and has been suggested to predict cardiovascular outcomes [41]. In humans, xanthosine can be produced from xanthosine monophosphate (XMP), and xanthosine may be catabolized to xanthine [42]. XMP is an intermediate molecule in IMP conversion to GMP [37]. Further supporting the relevance of the purine degradation pathway, xanthosine linked to glutamine in the case-to-control network comparison. Glutamine is an essential substrate in the synthesis of purines and pyrimidines [43], and the enzyme converting XMP to GMP requires glutamine in the process [44]. In the correlation networks, xanthosine correlated strongly with arabinose and allantoin. Allantoin, as the product of uric acid oxidative reactions, is considered a marker of oxidative stress in the human urine [45], suggesting xanthosine to indeed reflect purine degradation and oxidative stress. Targeting this purine degradation pathway, xanthine oxidase inhibitors have already been proposed as a putative drug target for CVD for individuals with related risk factors (e.g., diabetes) [39]. In summary, we hypothesize that the association between elevated xanthosine and increased risk of incident CAD reflects purine degradation, the oxidative stress induced by xanthine oxidoreductases, and the subsequent endothelial dysfunction.

trans-Aconitate was associated with decreased risk of 5-year incident CAD in type 1 diabetes, specifically among individuals with albuminuria. In the TCA cycle, the aconitase enzyme isomerizes citrate to isocitrate through cis-aconitate, while trans-aconitate is a damaged TCA cycle metabolite that inhibits the aconitase enzyme [46]. Therefore, trans-aconitate may cause accumulation of citrate, as proven at animal kidney cortex [47]. In line, we found trans-aconitate to correlate positively with citrate, even more so among individuals with incident CAD, while cis-aconitate was suggestively risk-increasingly associated with 10-year incident CAD. Urinary citrate has been associated with improved kidney function [48], and here, citrate was central throughout the network analysis, correlating positively with eGFR. trans-Aconitate itself did not correlate significantly with eGFR in the network analysis, suggesting an association with the 5-year CAD risk independent of glomerular filtration. However, trans-aconitate associated with 5-year incident CAD particularly in the presence of albuminuria. Importantly, plasma TCA cycle metabolic pathway has previously been associated with CAD [49]. TCA cycle disturbance could lead to mitochondrial dysfunction, subsequent oxidative stress and endothelial dysfunction [40, 50]. Endothelial dysfunction is essentially involved in CAD and predicts its adverse clinical outcomes [51]. Altogether our results support involvement of the TCA cycle metabolites in the development of CAD in diabetes.

Decreased 4-deoxythreonate, a secondary metabolite only recently identified in urine with NMR [52], was associated with 5-year incident CAD in type 1 diabetes. While little is known about 4-deoxythreonate, according to the network analysis, the 4-deoxythreonate association might relate to serum lipids, whereby dyslipidemia is a well-established risk factor for CAD both in diabetes and in the general population [53, 54]. In our cohort, 4-deoxythreonate correlated with 3-hydroxyisobutyrate. Of note, our recent genetic investigation for these 54 urine metabolites suggested that urine 4-deoxythreonate levels are affected by genes involved in neuron intrinsic apoptotic signaling pathway in response to oxidative stress [55].

Within the complete data network, sex was highly central, while many of the most central metabolites were associated with incident CAD in type 1 diabetes (*p* < 0.05). In fact, network centrality does imply physiological importance [56]. Within the network based on individuals with 10-year CAD and type 1 diabetes, the most central clinical variables were eGFR and triglycerides, which are well-known CAD risk factors [57, 58]. eGFR was central in the case-to-control correlation difference network, linking to eight metabolites. Thus, the influence of glomerular filtration on the urinary metabolome may be altered for individuals with up-coming CAD, which could have caused slight bias in the survival analysis even after adjustment for kidney function, especially for the protective metabolites given their stronger correlation with eGFR in general. There may be residual confounding, especially since eGFR is merely an estimation of glomerular filtration rate [59], while we did not consider the possibility of non-linearity in confounding.

Metabolomic state profiling implied that the urinary metabolites can predict CAD in type 1 diabetes (AUC_10-year/5-year_ = 0.655/0.670). However, metabolomic state profiling with only the CAD-associated metabolites (*p* < 0.05) predicted CAD in type 1 diabetes slightly worse, suggesting that some disease-causing effects remained undetected by the survival analysis. Modeling with the clinical variables outperformed modeling with the urinary metabolites (AUC_10-year/5-year_ = 0.792/0.791). Modeling with the CAD-associated metabolites in addition to the clinical variables did not significantly improve the prediction accuracy within the 10-year modeling scheme (AUC = 0.796, *p* = 0.63), but improved it significantly within the 5-year modeling scheme (AUC = 0.813, *p* = 0.044). Thus, the metabolites do capture short-term (i.e., 5-year) CAD-risk increasing effects independent of the conventional clinical variables. Despite the modesty in prediction improvement by urinary metabolites beyond the clinical variables, we hope that future efforts to build clinical risk scores for CAD in type 1 diabetes, especially short-term scores (e.g., 5-year), would consider the potential benefits of the urinary metabolites as non-invasive biomarkers providing additional information on CAD. In addition, small improvements in predictive performance can be clinically meaningful [60].

This study has limitations. First, the reported urinary metabolite associations were not replicated in an independent cohort due to lack of suitable large cohorts. To our knowledge, we have here performed the first large-scale prospective urinary metabolomics analysis on CAD in diabetes. Therefore, although external replication is most definitely needed for the reported metabolites, this shall be reliant on future efforts when data from other cohorts will be available. Accordingly, more research is needed to elaborate the cohort-specificity in the presented network analysis and metabolomic state profiling. In addition, despite the prospective study setting and careful covariate adjustment, we cannot infer causality for the associations. Furthermore, we studied only a small subset of the true urinary metabolome, i.e., 54 metabolites, while 5660 metabolites have been reported in the human urine (*hmdb.ca*). In fact, some urinary metabolites, which were not studied here, are likely to be associated with incident CAD in type 1 diabetes and could be measured with non-targeted urinary metabolomics, e.g., with mass-spectrometry based methods. The current urine NMR platform was chosen as a compromise between affordability allowing a larger study cohort, and thus, higher statistical power to discover novel associations, and the number of captured metabolites, but still covering a broad spectrum of metabolic pathways. However, metabolomic state profiling with more urinary metabolites would likely outperform the predictive model presented in this study. The network analysis entails a specific limitation; the reported centrality partly reflects the potential centrality across the 54 metabolites and not the true metabolome centrality. Finally, the relationship between urinary metabolites and CAD is not extensively researched in the general population. Therefore, to establish diabetes-specificity of our results, similar studies are needed in the general population as well. Strengths of this study include a large and well-characterized cohort, although with case–control imbalance, and comprehensively performed analyses.

## Conclusions

We performed the first large-scale urinary metabolomic analysis for CAD in type 1 diabetes and found decreased 3-hydroxyisobutyrate and increased xanthosine to associate with 10-year incident CAD, as well as decreased 4-deoxythreonate and decreased trans-aconitate to associate with 5-year incident CAD, beyond clinical risk factors. We hypothesize that the 3-hydroxysiobutyrate association with incident CAD in type 1 diabetes reflects improved kidney function and younger age, and the xanthosine association with incident CAD in type 1 diabetes to reflect purine degradation and the following oxidative stress. Furthermore, glomerular filtration rate affected the urinary metabolome differently in individuals with and without incident CAD. Altogether, we demonstrated that urinary metabolites may be utilized for CAD prediction in type 1 diabetes. The reported metabolite-associations suggest novel molecular pathways involved in CAD pathophysiology in type 1 diabetes, and hold promise for improving CAD prediction in type 1 diabetes.

## Supplementary Information

Additional file 1.

Additional file 2.

## Data Availability

The individual-level data analyzed here is not publicly available due to study consent provided by the participants at the time of data collection, but readers may propose collaboration with the corresponding author.

## Abbreviations

AGEs: Advanced glycation end-products
AHT: Antihypertensive treatment
CAD: Coronary artery disease
CVD: Cardiovascular disease
BMI: Body mass index
DBP: Diastolic blood pressure
DKD: Diabetic kidney disease
eGDR: Estimated glucose disposal rate
eGFR: Estimated glomerular filtration rate
FinnDiane: Finnish Diabetic Nephropathy
HbA_1c_: Glycated hemoglobin
HDL: High density lipoprotein
KRT: Kidney replacement therapy
LDL: Low density lipoprotein
MAP: Mean arterial pressure
NMR: Nuclear magnetic resonance
SBP: Systolic blood pressure
SVM: Support vector machine
TCA: Tricarboxylic acid cycle
WHtR: Waist-to-height ratio
XOR: Xanthine oxidoreductase
