# Supplementary material for: Urinary metabolomics provide insights into coronary artery disease in individuals with type 1 diabetes
Source: Cardiovasc Diabetol. 2024 Nov 26;23:425. doi: 10.1186/s12933-024-02512-8 (PMC11590341; doi:10.1186/s12933-024-02512-8)
Supplement: Supplementary file 1 — Additional file 1. [file 12933_2024_2512_MOESM1_ESM.docx]

Supplementary material

**Urinary metabolomics provide insights into coronary artery disease in individuals with type 1 diabetes**
Anni A Antikainen, Stefan Mutter, Valma Harjutsalo, Lena M Thorn, Per-Henrik Groop, Niina Sandholm, on behalf of the FinnDiane Study Group

[Supplementary Figures 1](#_Toc180762600)

[**Fig. S1** Patient selection flow chart. 1](#_Toc180762601)

[**Fig. S2** Baseline clinical characteristics in urine metabolomics (N=2,501). 2](#_Toc180762602)

[**Fig. S3** Detailed baseline clinical characteristics in urine metabolomics (N=2,501). 3](#_Toc180762603)

[**Fig. S4** Standardized urine metabolite-to-creatinine ratios (N=2,501). 4](#_Toc180762604)

[**Fig. S5** Principal component analysis of the urine metabolome. 9](#_Toc180762605)

[**Fig. S6** Metabolic profiling model training scheme within one bootstrap. 10](#_Toc180762606)

[**Fig. S7** Metabolite’s incident CAD survival hazard ratio with respect to the metabolite’s correlation with eGFR. 11](#_Toc180762607)

[**Fig. S8** Time-dependency of metabolite-CAD effect size for metabolite-to-creatinine ratios displaying non-proportional hazards (*p*<0.05) in 10-year CAD survival models. 12](#_Toc180762608)

[**Fig. S9** Metabolome-wide survival analysis with the full follow-up survival modeling (N=2,953). 13](#_Toc180762609)

[**Fig. S10** Dimethylamine (**A**) and uracil (**B**) quartile’s survival probability within the full follow-up coronary artery disease risk survival models. 14](#_Toc180762610)

[**Fig. S11** Complete baseline correlation coefficient network (*p*<2×10^-5^, N_patient_=2,501). 15](#_Toc180762611)

[**Fig. S12** Cases’ baseline correlation coefficient network (*p*<2×10^-5^, N_patient_=209). 16](#_Toc180762612)

[**Fig. S13** Correlation coefficient differences (*p*_difference_<0.05, *p*_case or control_<2×10^-5^). 17](#_Toc180762613)

[**Fig. S14** Local network characteristics of the case-to-control correlation difference network. 20](#_Toc180762614)

[**Fig. S15** 10-year coronary artery disease (CAD) prediction with support vector machine in each bootstrap. 21](#_Toc180762615)

[**Fig. S16** 5-year coronary artery disease (CAD) prediction with support vector machine in each bootstrap. 22](#_Toc180762616)

[Supplementary Tables 23](#_Toc180762617)

[**Table S1** Coronary artery disease definition with registry data. 23](#_Toc180762618)

[**Table S2** Baseline clinical characteristics for cases who developed coronary artery disease (CAD) and controls who did not during the full follow-up (N=2,953, mean ± SD). 24](#_Toc180762619)

[**Table S3** Urine metabolites. Detection limit, percent of missing values within the data (%), number of metabolite measurements replaced with detection limit (N), and distribution transformation method. 25](#_Toc180762620)

[**Table S4** Baseline clinical characteristics for cases who developed coronary artery disease (CAD) and controls who did not, without (**A**) and with (**B**) albuminuria, during the 10-year follow-up (N=2,501, mean ± SD). 27](#_Toc180762621)

[**Table S5** 3-hydroxyisobutyrate (3-HIB) and estimated glucose disposal rate (eGDR). 29](#_Toc180762622)

[**Table S6** 5-year coronary artery disease (CAD) risk models for metabolites displaying effect size time-dependency in the main analysis with a specific adjustment setting (N=2,501). 30](#_Toc180762623)

[**Table S7** 5-year coronary artery disease (CAD) risk models for urine metabolites displaying effect size time-dependency in the albuminuria status specific sub-analyses. 31](#_Toc180762624)

[**Table S8** Global properties of the 10-year coronary artery disease data metabolic networks (N=2,501). 32](#_Toc180762625)

[**Table S9** Xanthosine and 3-hydroxyisobutyrate absolute concentration (mmol/l) association with clinical data. 33](#_Toc180762626)

[**Table S10** FinnDiane study centers. 34](#_Toc180762627)

# Supplementary Figures

**Fig. S1** Patient selection flow chart. NMR: Nuclear magnetic resonance, Duration: Diabetes duration, CAD: Coronary artery disease, KRT: Kidney replacement therapy, HF: Heart failure. Figure created with BioRender.com.


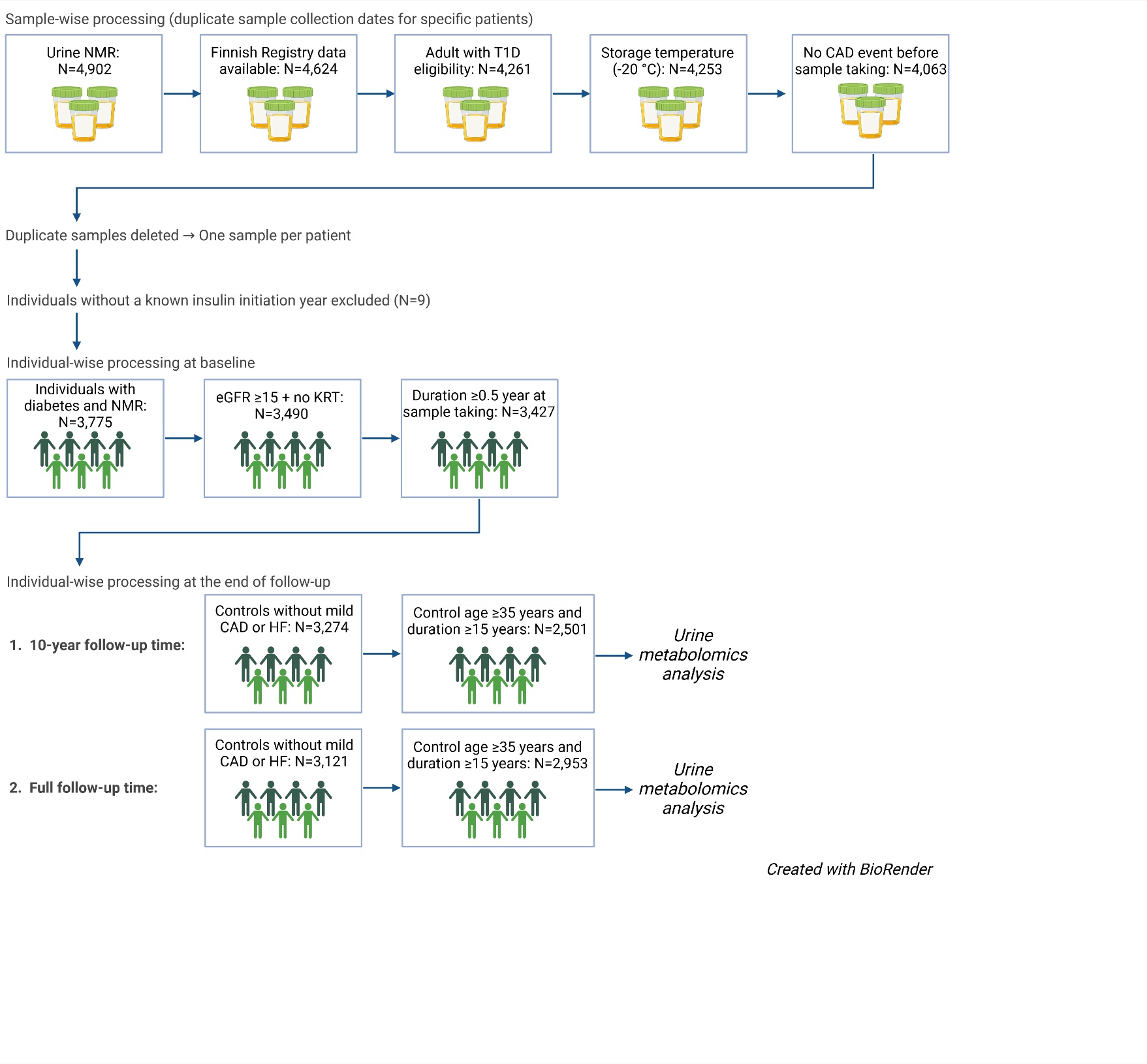


## **Fig. S2** Baseline clinical characteristics in urine metabolomics (N=2,501).


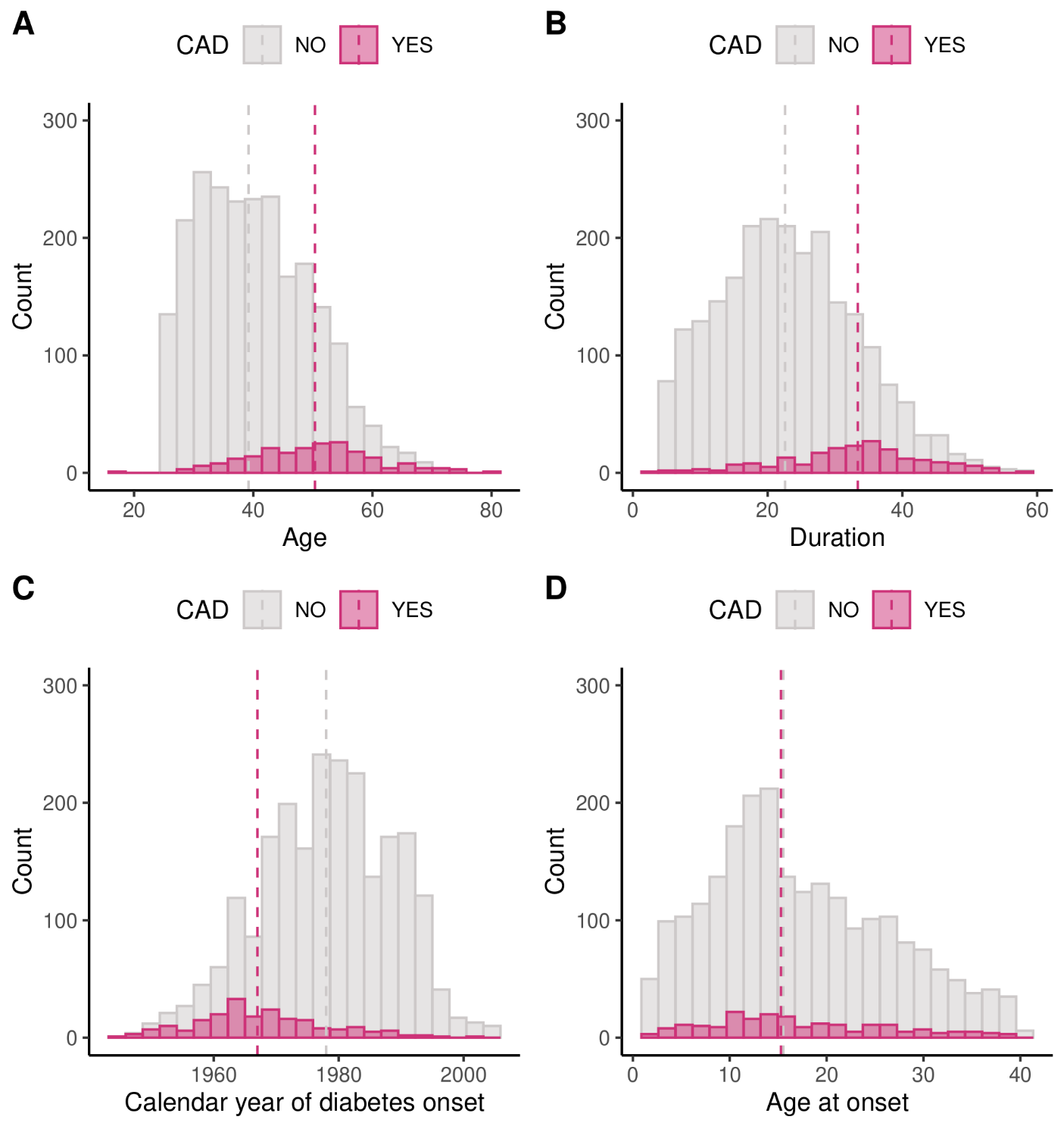


## **Fig. S3** Detailed baseline clinical characteristics in urine metabolomics (N=2,501).


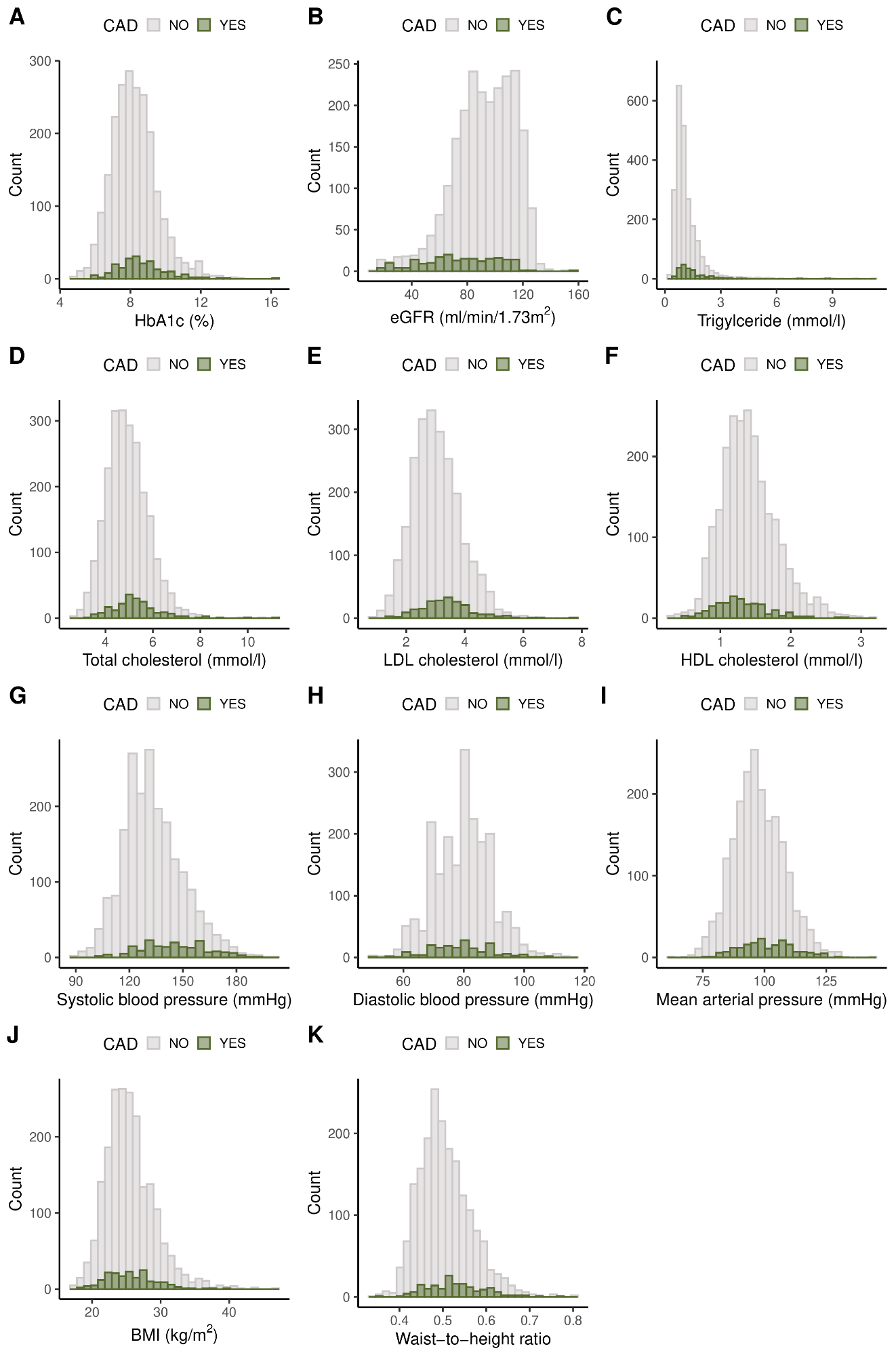


## **Fig. S4** Standardized urine metabolite-to-creatinine ratios (N=2,501).

**
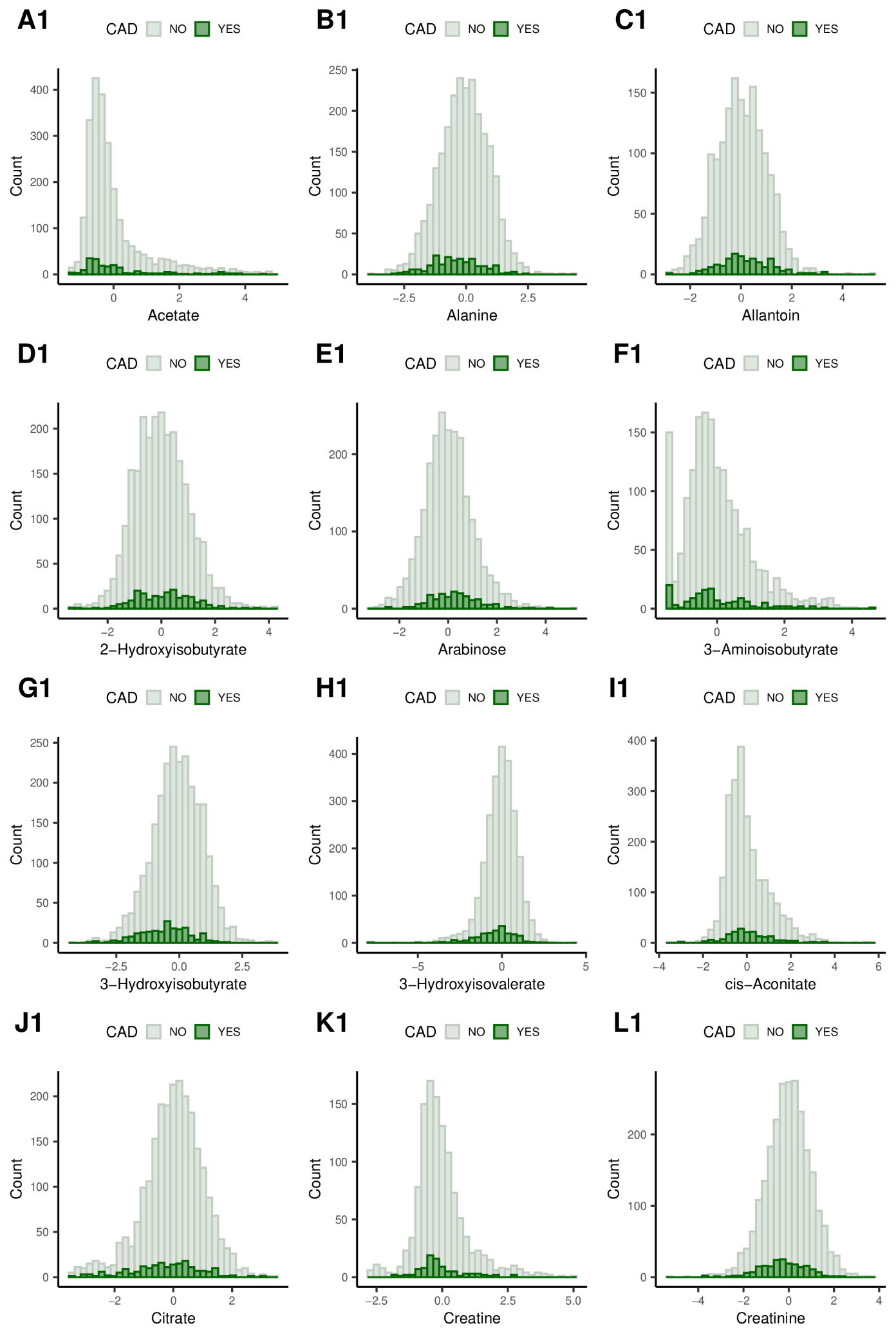
**

**
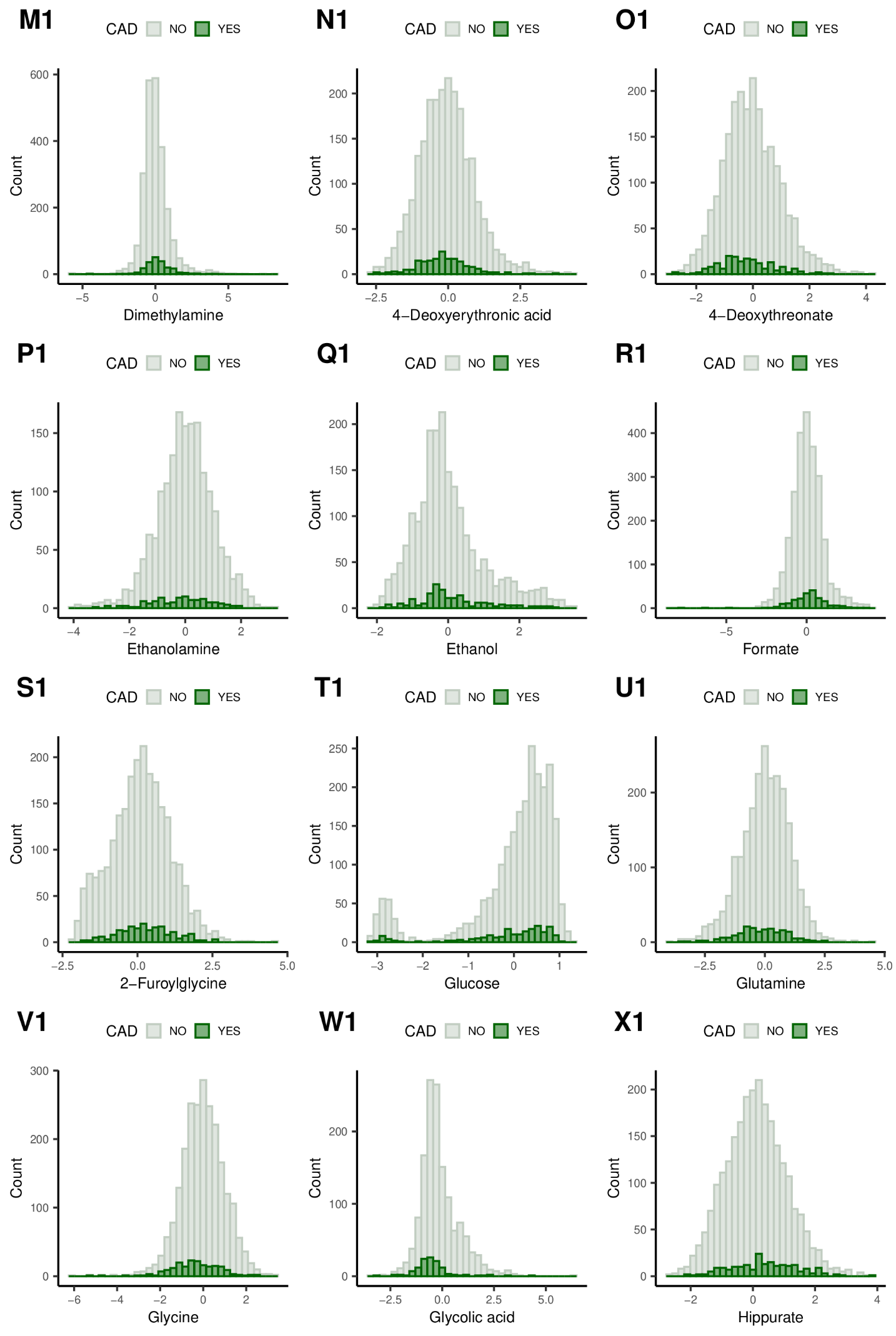
**

**
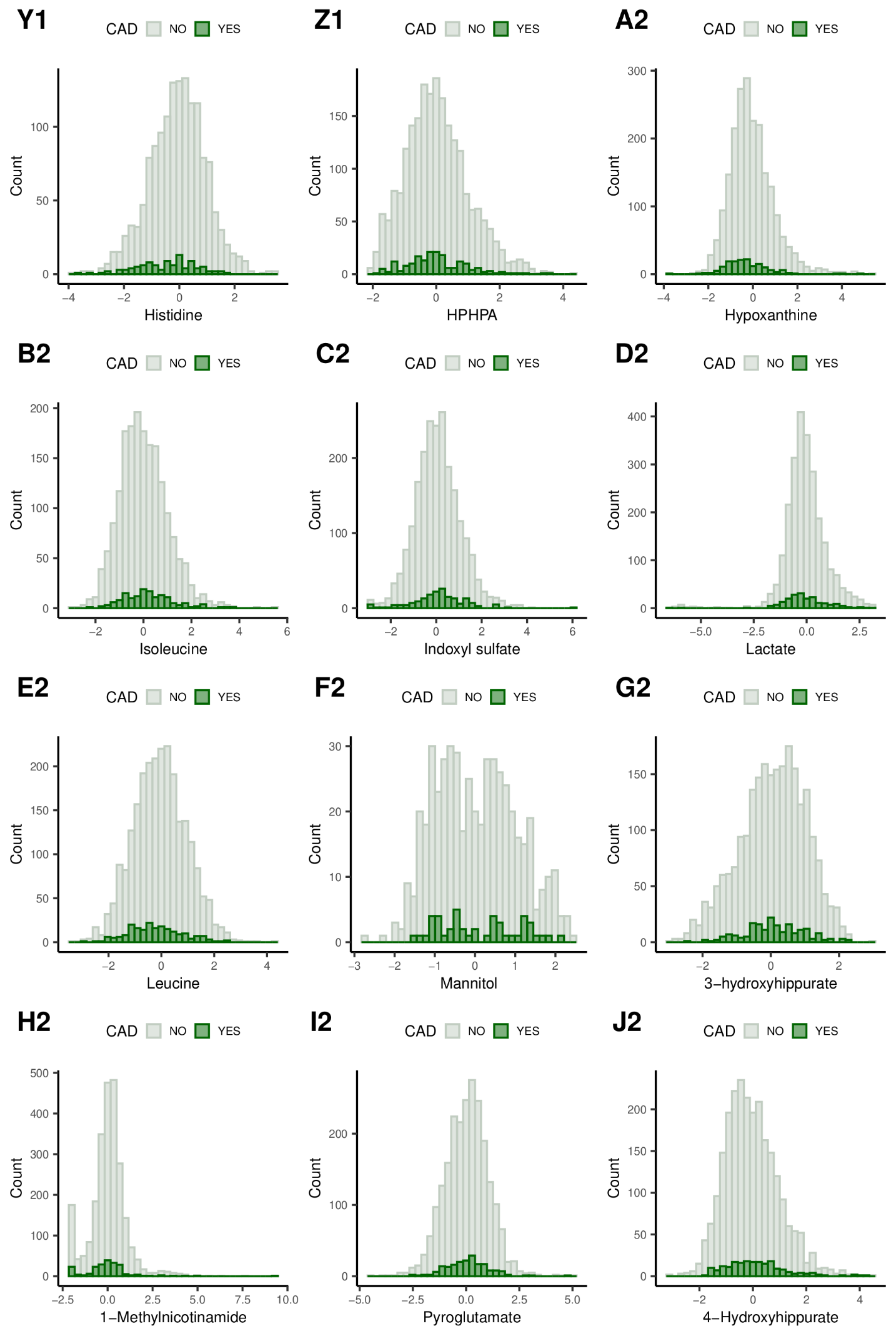
**

**
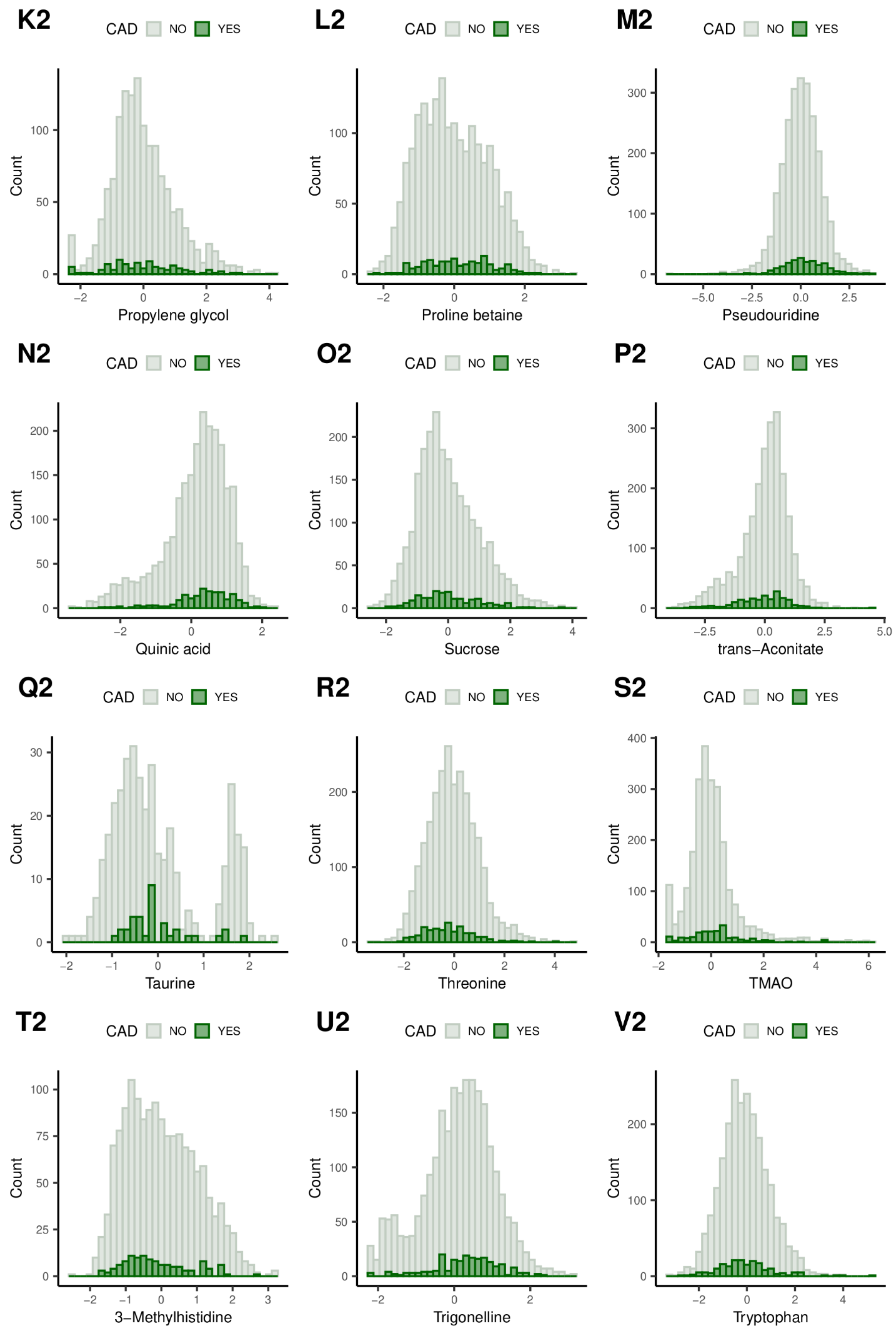
**

**
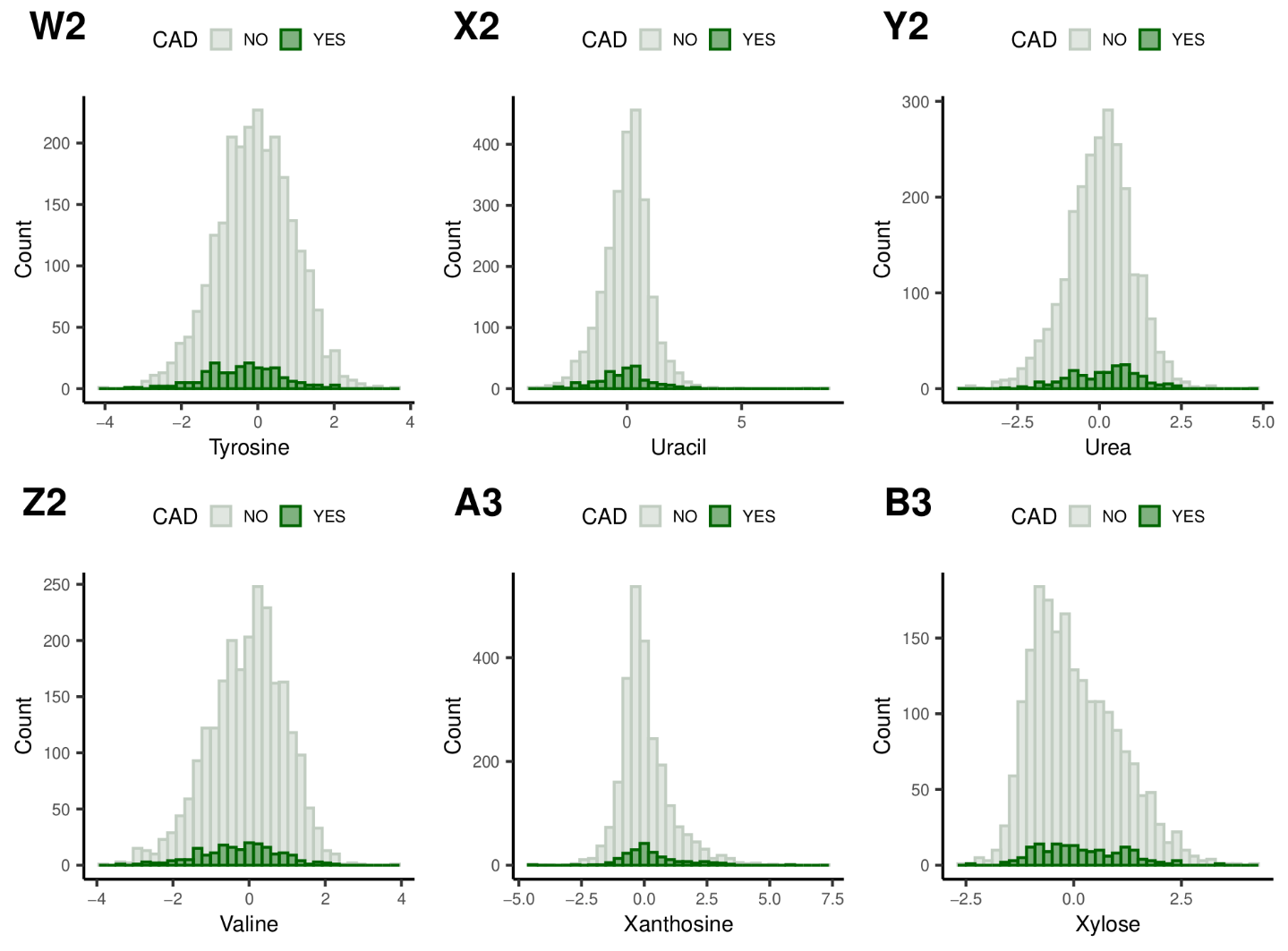
**

## **Fig. S5** Principal component analysis of the urine metabolome.


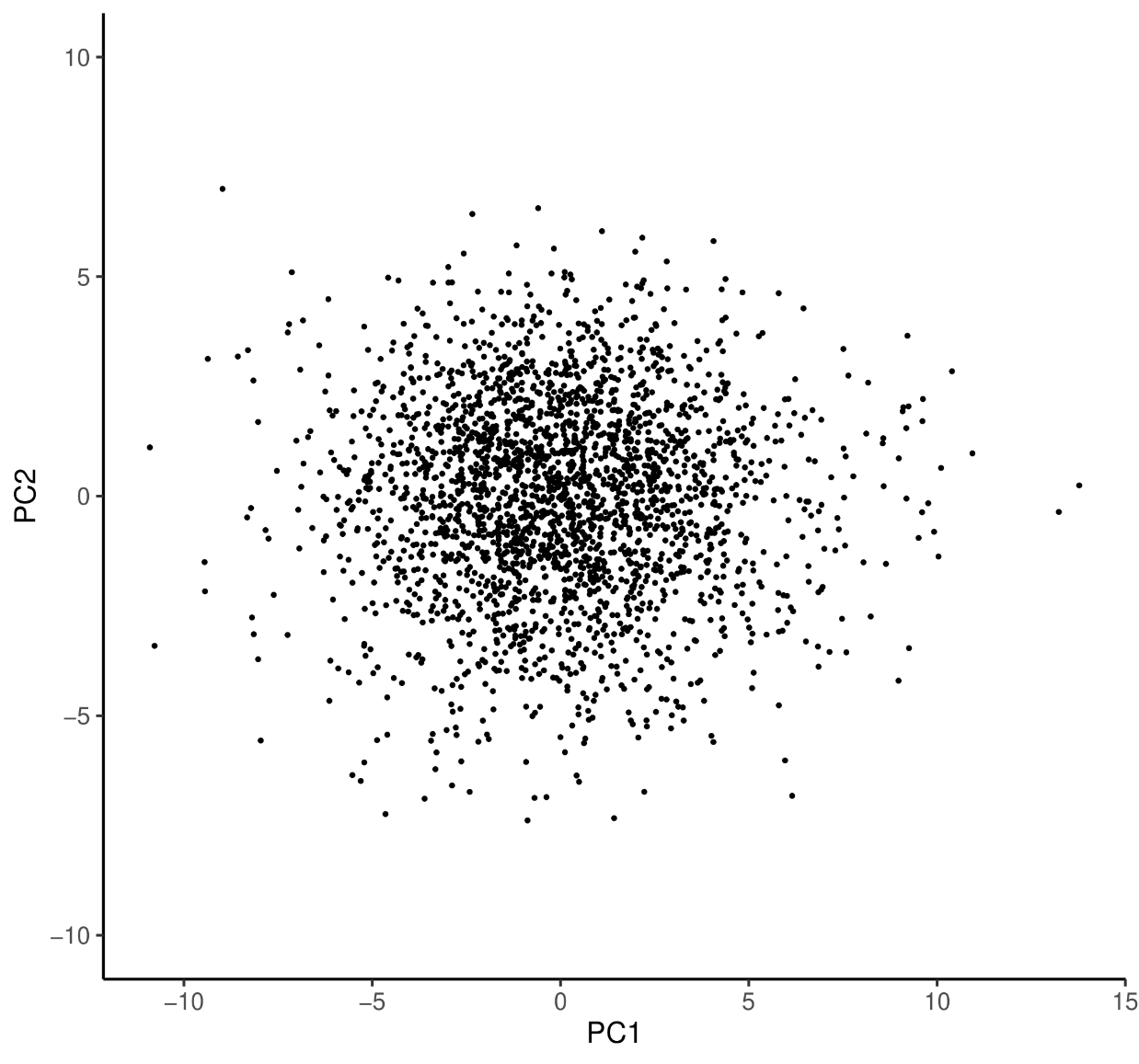


**Principal component analysis (PCA):** Three metabolites were excluded from the PCA analysis due to >50% missingness rate (taurine, creatine, mannitol); while missing values of the remaining metabolites were replaced by the corresponding metabolite’s distribution average. Forty-six metabolites explained roughly 99% of variance in the complete data (N=51). For calculation of multiple testing correction level, we included the excluded three metabolites (N=3: taurine, creatine, mannitol) to the number of metabolites explaining 99% of variance in the data (N=46) and corrected the significance threshold by the number of these metabolites (N=46+3=49). Thus, we considered the threshold, *p*=0.05/49=0.00102, as the multiple testing corrected significance threshold throughout the 10-year CAD risk metabolome-wide survival analyses.

**Fig. S6** Metabolic profiling model training scheme within one bootstrap. The presented modeling scheme is repeated in 10 bootstraps from which results are presented separately. Modeling includes pre-training for variable selection, and support vector machine (SVM) training with bagging (metabolic profiling), and model validation. Figure created with BioRender.com.


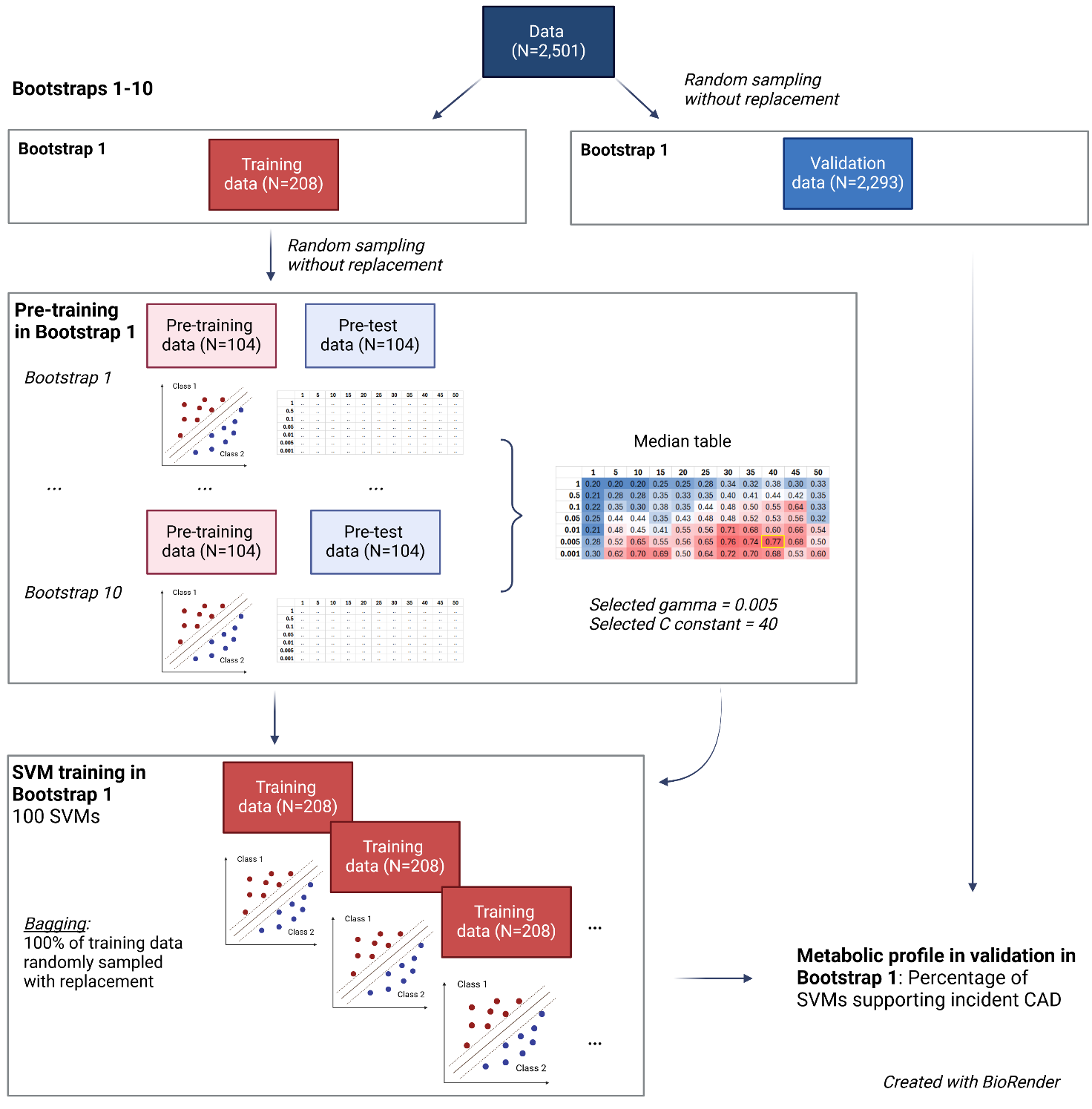


**Fig. S7** Metabolite’s incident CAD survival hazard ratio with respect to the metabolite’s correlation with eGFR. **A.** Survival model with adjustment for non-modifiable risk factors, **B.** Survival model with adjustment for non-modifiable risk factors and kidney function, **C.** Survival model with adjustment for non-modifiable risk factors, kidney function and established CAD risk factors. Metabolites significant after multiple testing are highlighted in the figures (*p*<0.00102).


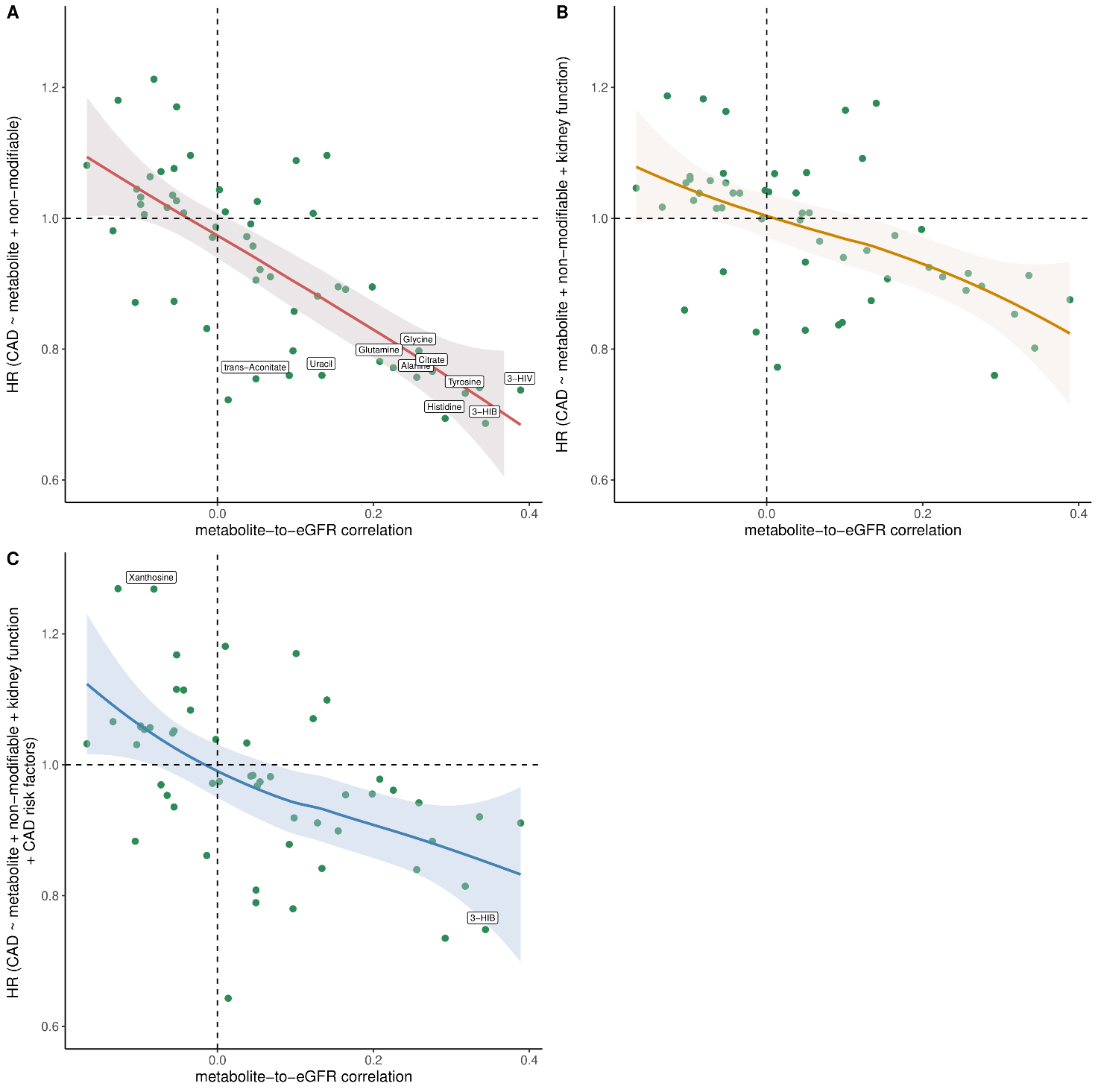


## **Fig. S8** Time-dependency of metabolite-CAD effect size for metabolite-to-creatinine ratios displaying non-proportional hazards (*p*<0.05) in 10-year CAD survival models.

| 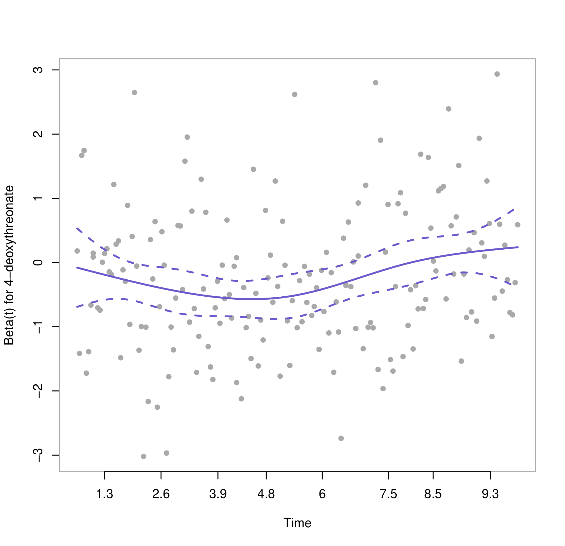 | 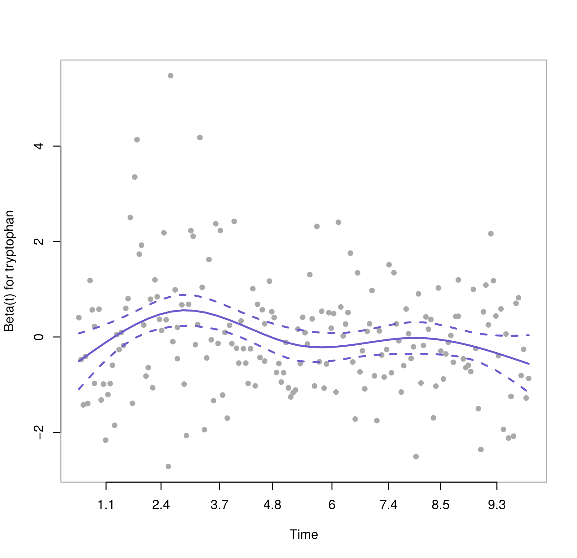 |
| --- | --- |
| 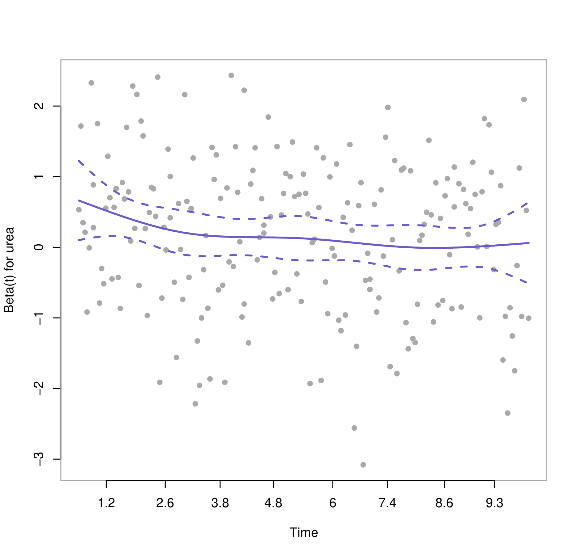 | 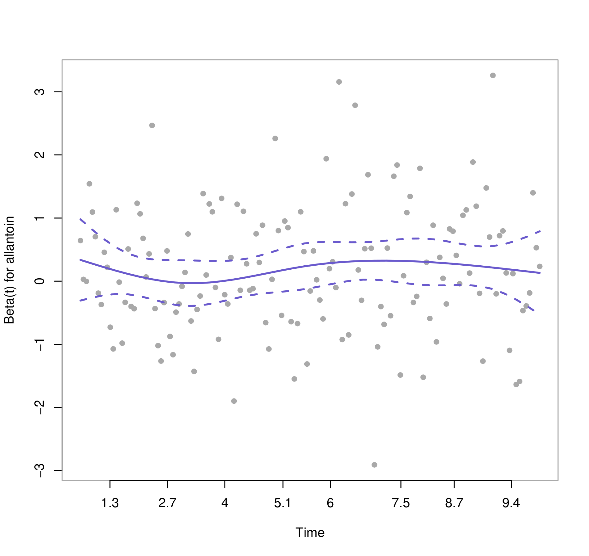 |
| 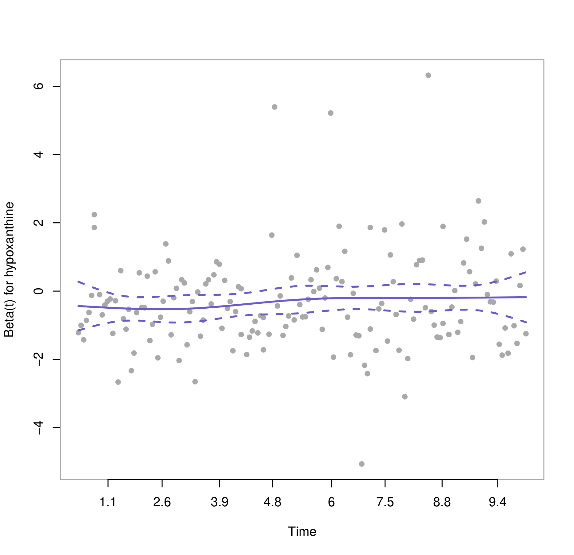 | 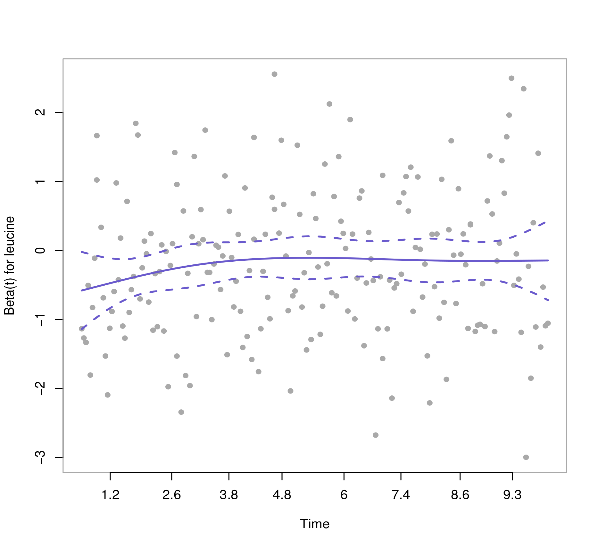 |
| 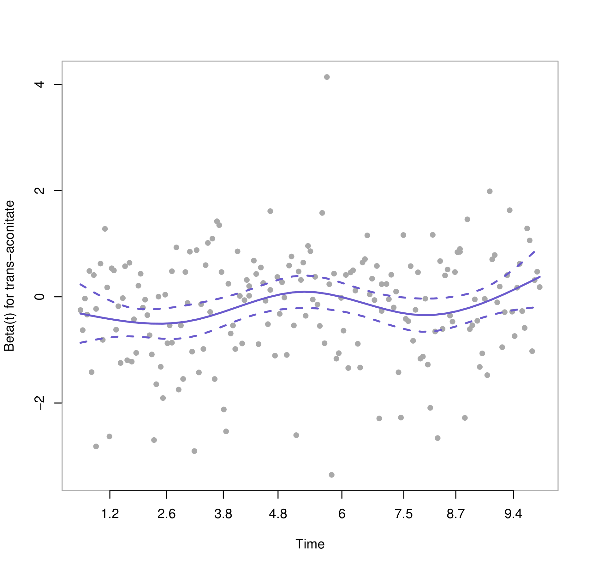 |  |

**Fig. S9** Metabolome-wide survival analysis with the full follow-up survival modeling (N=2,953). Survival analyses with three adjustment settings: **1.** Non-modifiable risk factor (age, sex, diabetes onset calendar year), **2.** Non-modifiable risk factor + kidney function (eGFR, albuminuria), **3.** Non-modifiable risk factor + kidney function + coronary artery disease (CAD) risk factor (systolic blood pressure, LDL cholesterol, waist-to-height ratio, HbA_1c_, smoking). Figure displays significant metabolite-CAD associations (*p*<0.05); and dark color indicates metabolome-wide significance (*p*<0.00102). Metabolites displaying time-dependency in non-adjusted models were excluded; and we considered 37 urinary metabolites. TMAO: trimethylamine N-oxide.


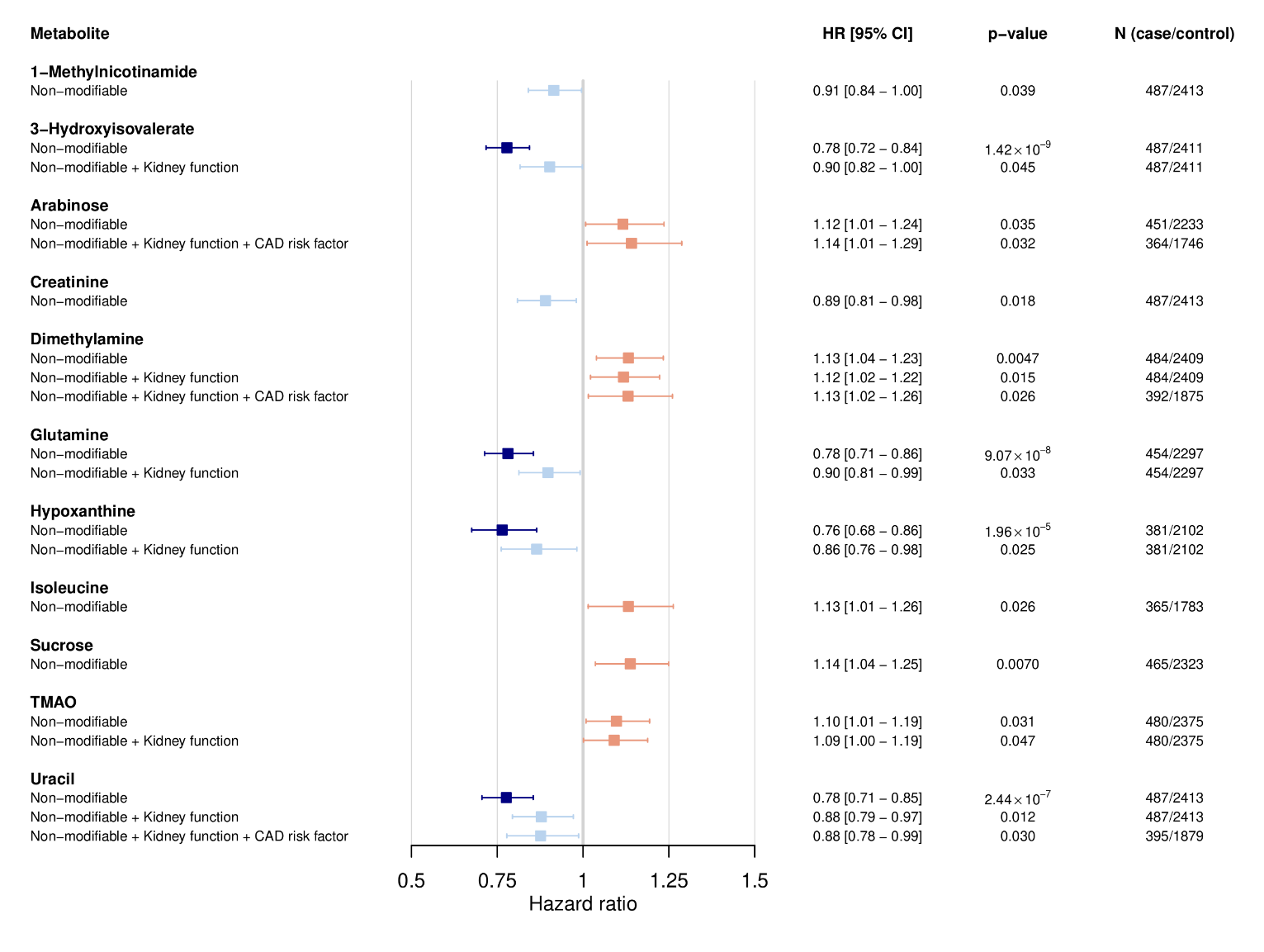


**Fig. S10** Dimethylamine (**A**) and uracil (**B**) quartile’s survival probability within the full follow-up coronary artery disease risk survival models. Adjusted for age, sex, diabetes onset calendar year, eGFR, albuminuria, HbA_1c_, systolic blood pressure, LDL cholesterol, waist-to-height ratio, and smoking (N=2,953).


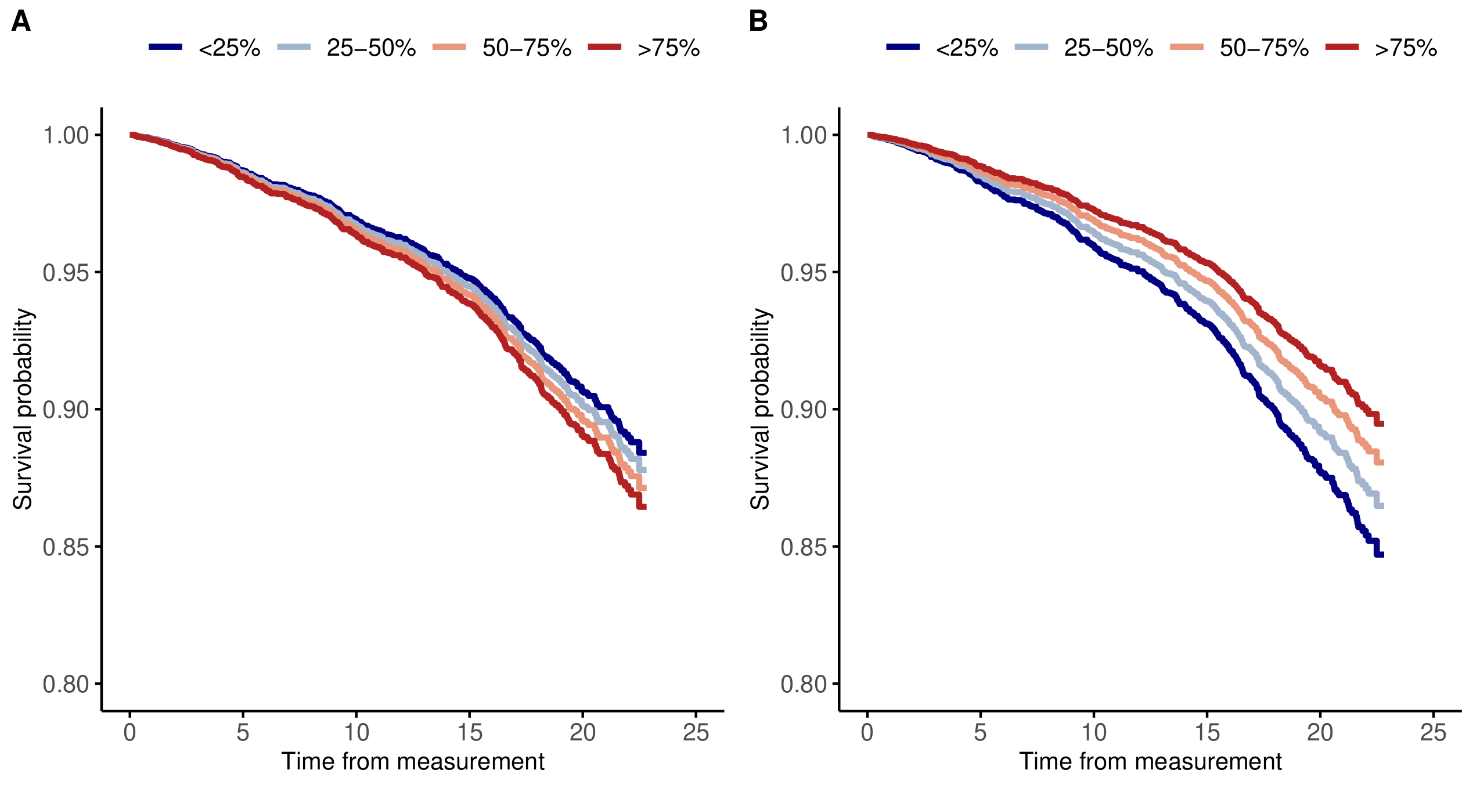


**Fig. S11** Complete baseline correlation coefficient network (*p*<2×10^-5^, N_patient_=2,501). Clinical variable nodes are colored as beige and metabolite nodes as pink, darker pink for metabolites associated with 10-year incident coronary artery disease (CAD) (*p*<0.05). Red links represent positive correlation coefficient, and blue links negative, and the link weight represent the magnitude. 4-Hydroxyhippurate (4-HH), dimethylamine (DMA), trimethylamine N-oxide (TMAO), 2-hydroxyisobutyrate (2-HIB), 3-hydroxyhippurate (3-HH), 3-(3-hydroxyphenyl)-3-hydroxypropionic acid (HPHPA), 3-aminoisobutyrate (3-AIB), 1-methylnicotinamide (1-MNA), 4-deoxyerythronic acid (4-DEA), 3-methylhistidine (3-MH), 4-deoxythreonate (4-DTA), 3-hydroxyisovalerate (3-HIV), 3-hydroxyisobutyrate (3-HIB), waist-to-height ratio (WHtR), triglyceride (TG), systolic blood pressure (SBP), diastolic blood pressure (DBP), mean arterial pressure (MAP).


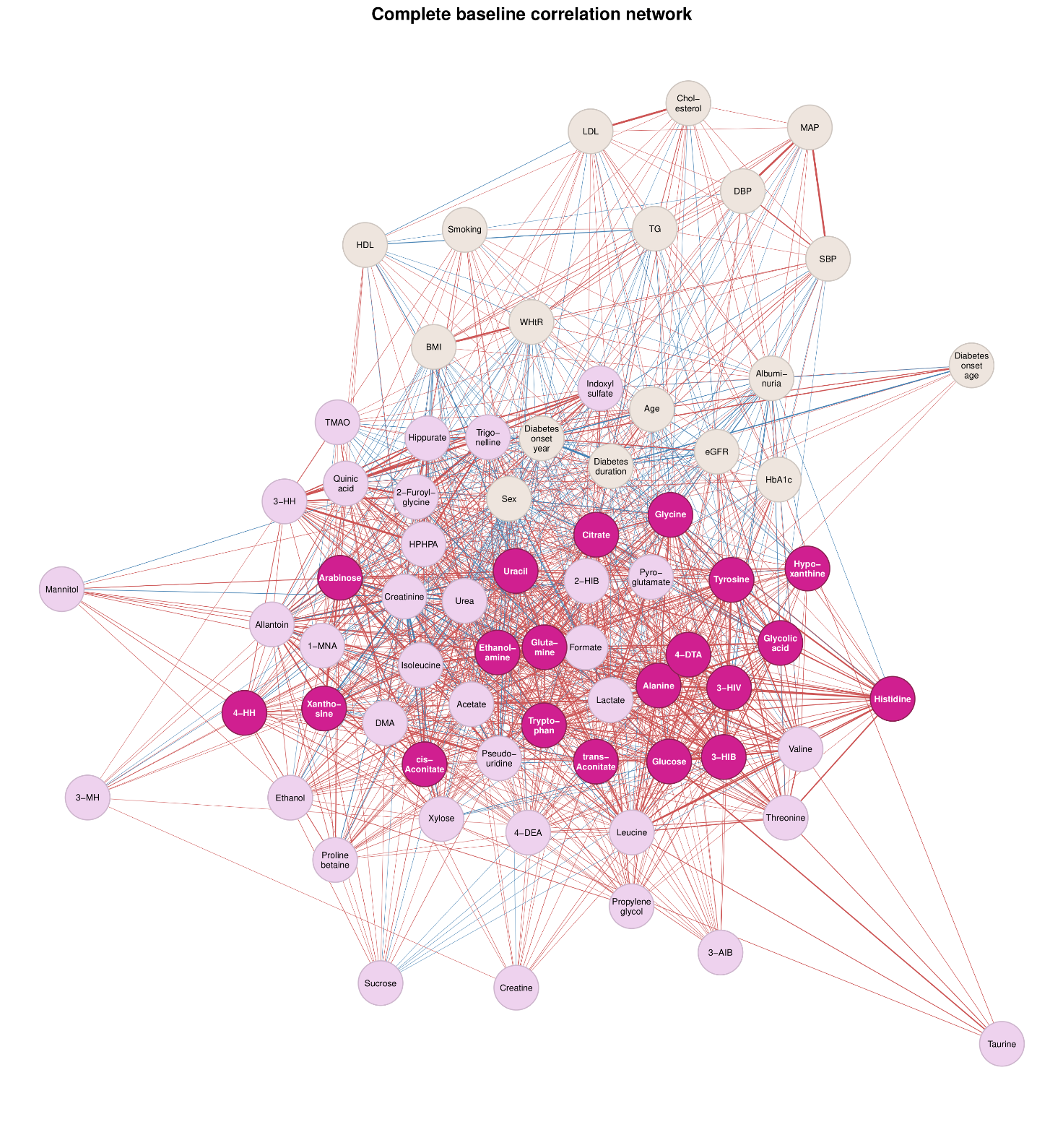


**Fig. S12** Cases’ baseline correlation coefficient network (*p*<2×10^-5^, N_patient_=209). Clinical variable nodes are colored as beige and metabolite nodes as pink, darker pink for metabolites associated with 10-year incident coronary artery disease (CAD) (*p*<0.05). Red links represent positive correlation coefficient, and blue links negative, and the link weight represent the magnitude. 4-Hydroxyhippurate (4-HH), dimethylamine (DMA), trimethylamine N-oxide (TMAO), 2-hydroxyisobutyrate (2-HIB), 3-hydroxyhippurate (3-HH), 3-(3-hydroxyphenyl)-3-hydroxypropionic acid (HPHPA), 3-aminoisobutyrate (3-AIB), 1-methylnicotinamide (1-MNA), 4-deoxyerythronic acid (4-DEA), 3-methylhistidine (3-MH), 4-deoxythreonate (4-DTA), 3-hydroxyisovalerate (3-HIV), 3-hydroxyisobutyrate (3-HIB), waist-to-height ratio (WHtR), triglyceride (TG), systolic blood pressure (SBP), diastolic blood pressure (DBP), mean arterial pressure (MAP).


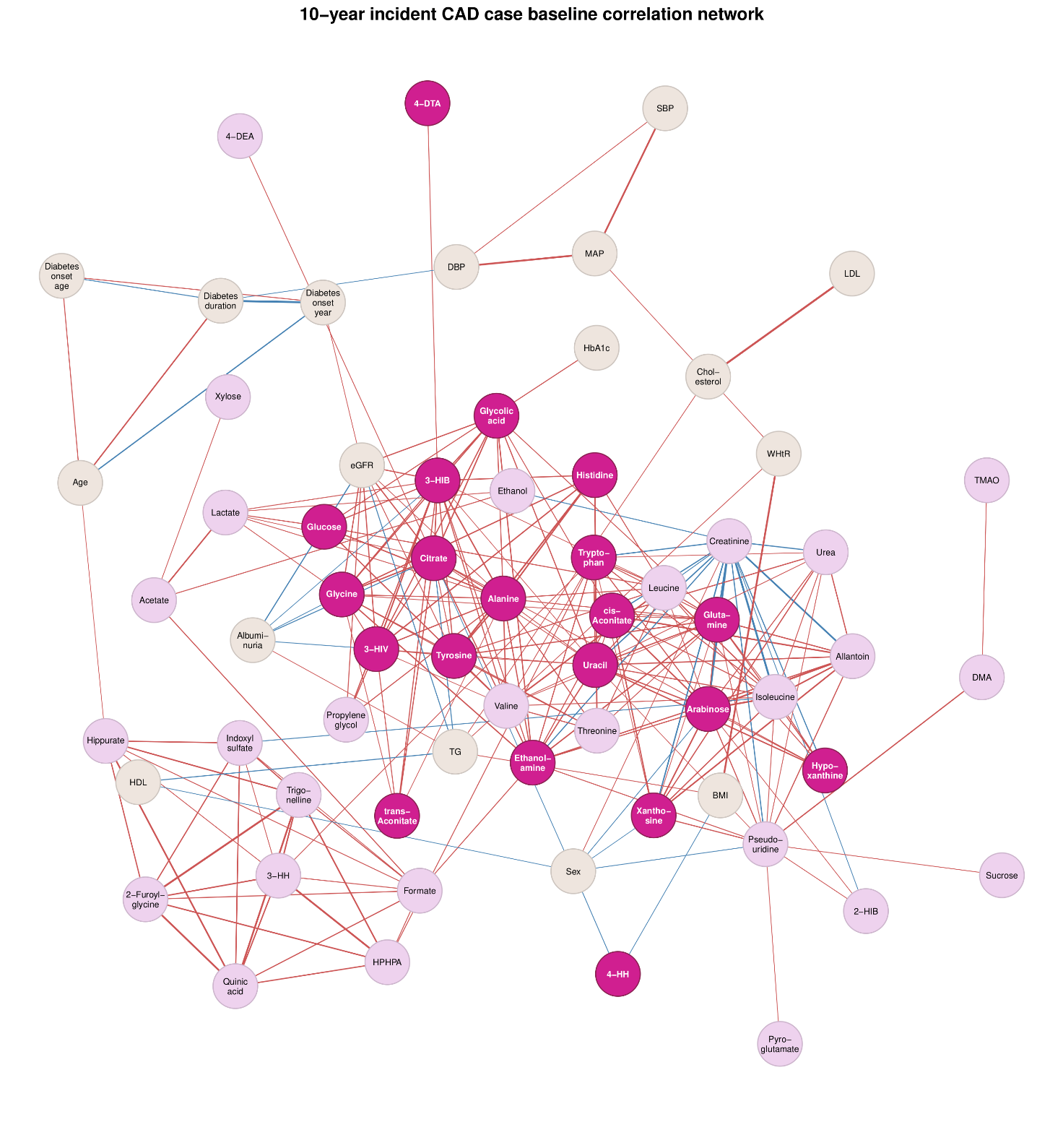


**Fig. S13** Correlation coefficient differences (*p*_difference_<0.05, *p*_case or control_<2×10^-5^). Correlation difference between those with and without 10-year incident CAD (**A**), correlation in individuals with (**B**), and without the CAD (**C**). 3-(3-hydroxyphenyl)-3-hydroxypropionic acid (HPHPA). +Association is non-significant (*p*>2×10^-5^) in networks (**B**) or (**C**).

| **A** | **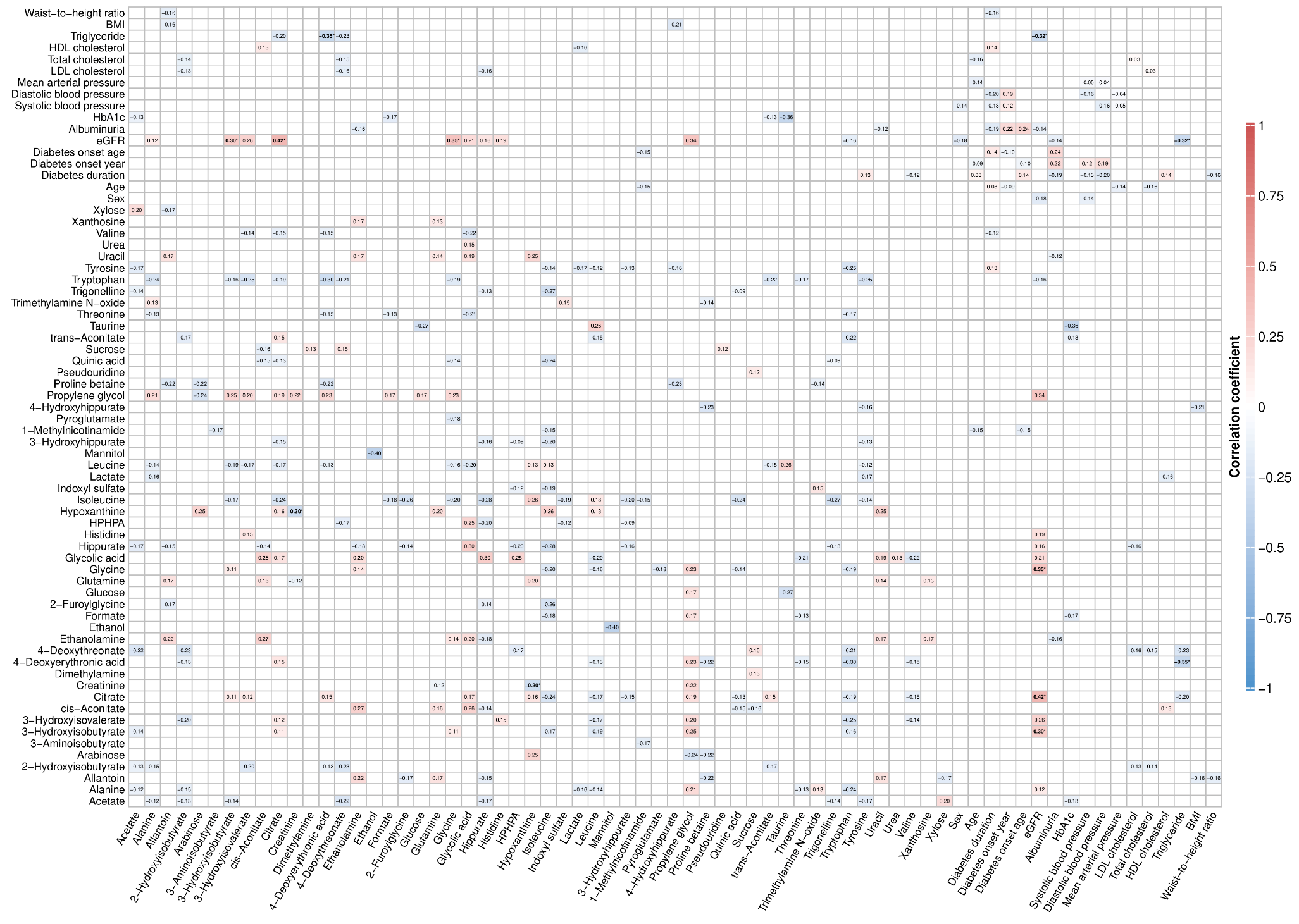** |
| --- | --- |
| **B** | **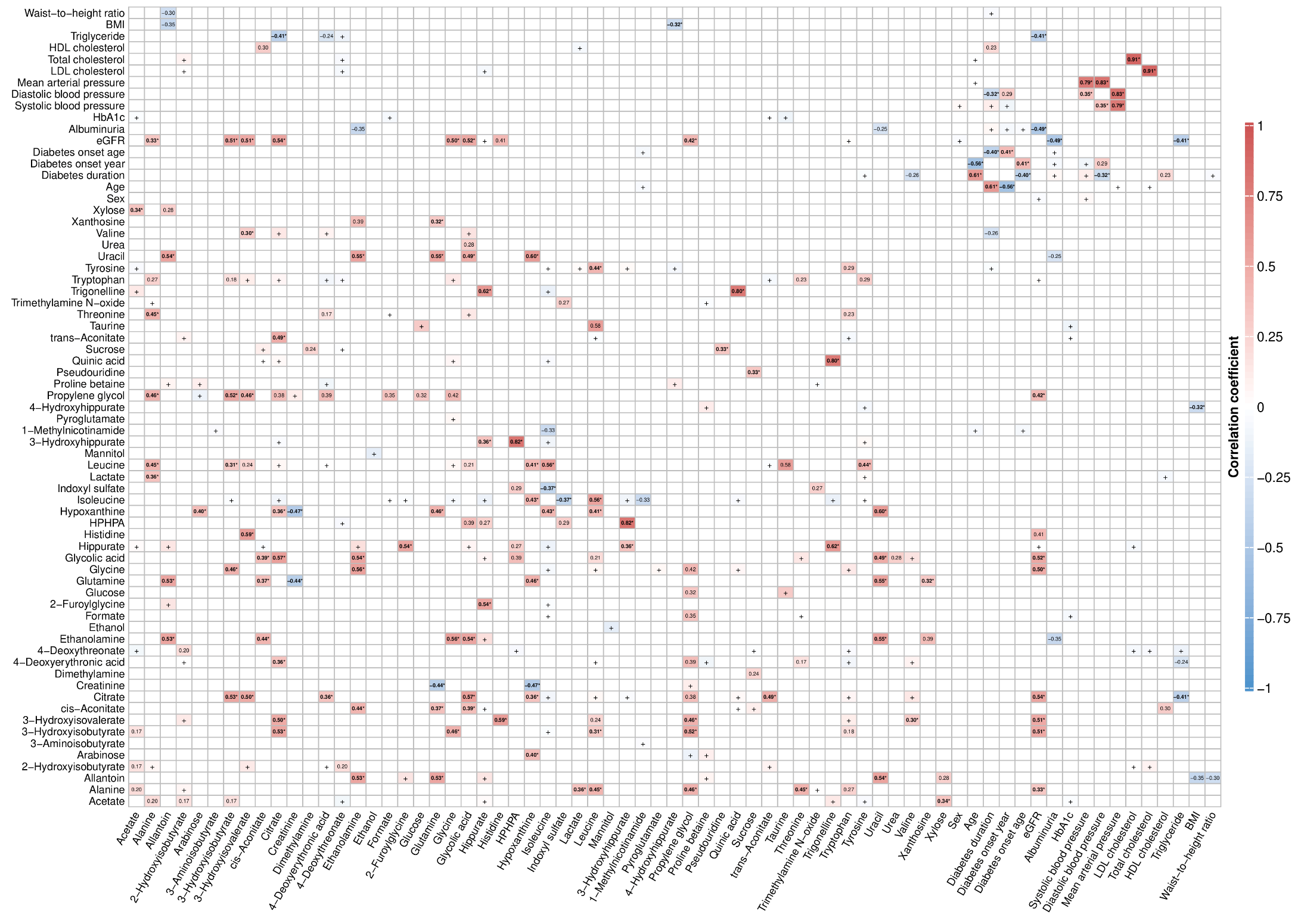** |
| **C** | **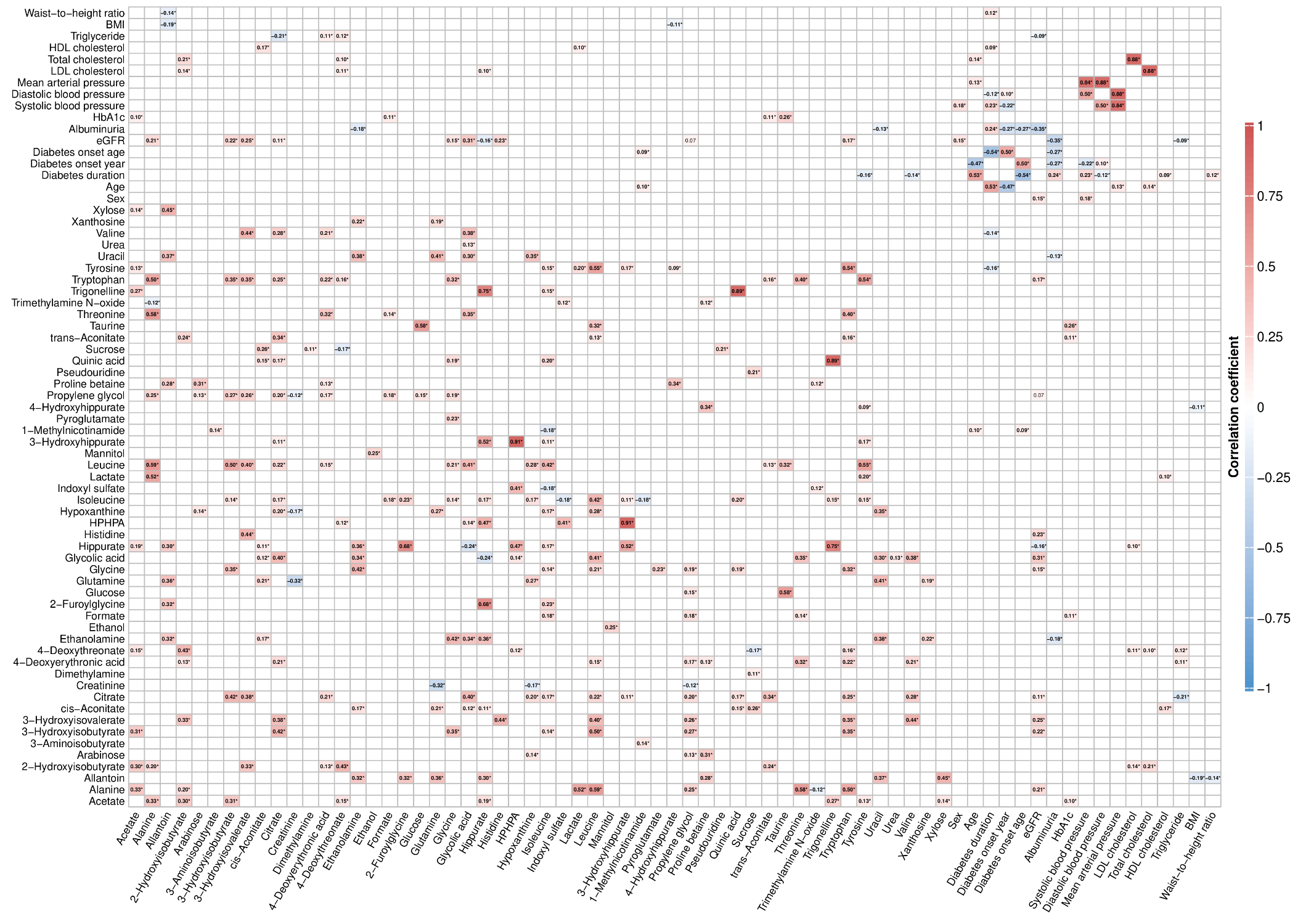** |

**Fig. S14** Local network characteristics of the case-to-control correlation difference network. Metabolites, which were associated with 10-year incident coronary artery disease (*p*<0.05) are highlighted with pink. HPHPA: 3-(3-hydroxyphenyl)-3-hydroxypropionic acid.


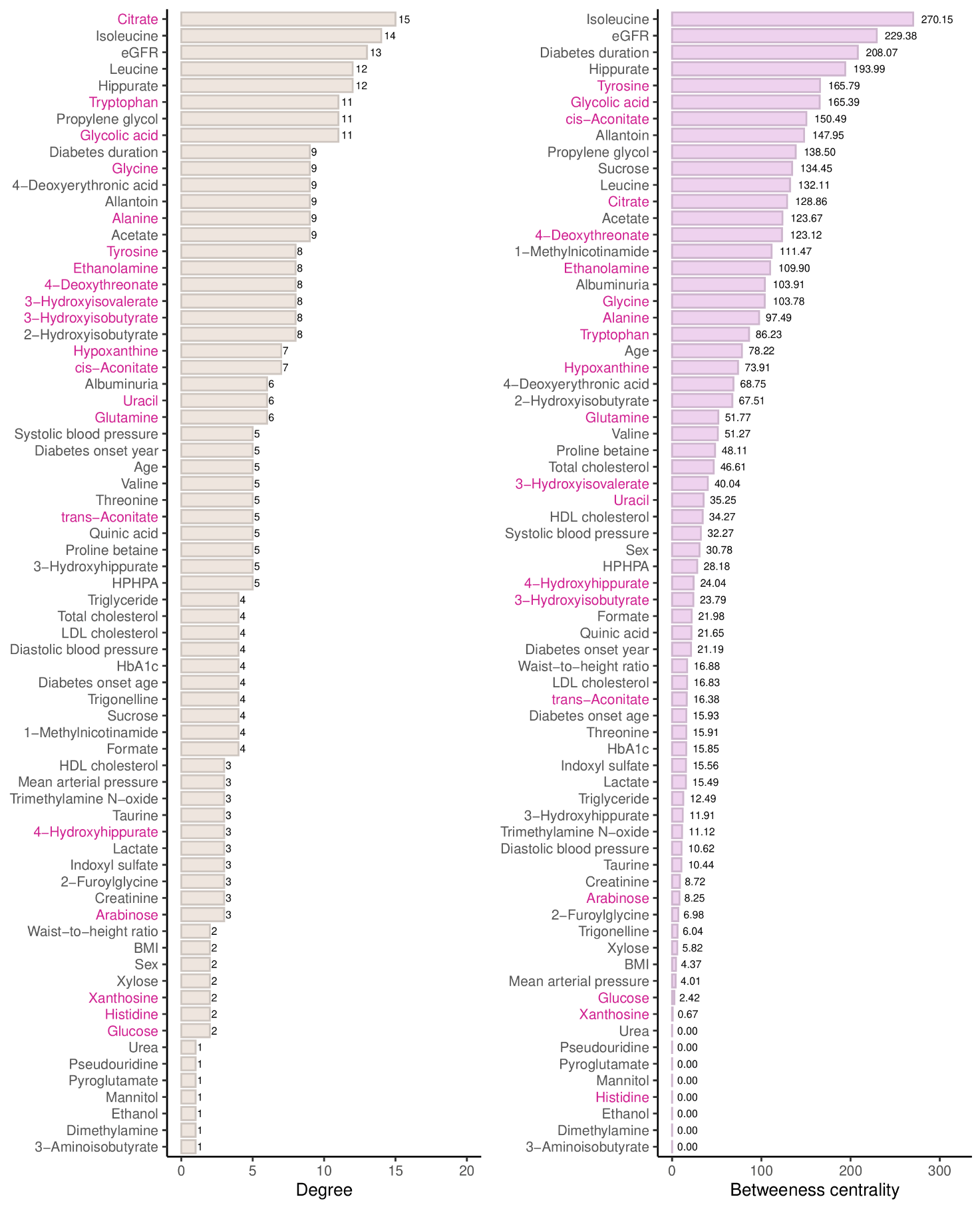


**Fig. S15** 10-year coronary artery disease (CAD) prediction with support vector machine in each bootstrap. **A.** Prediction accuracy (AUC), **B.** Optimal prediction cutoff from the percentage score (0-1) with Youden method from ROC curve. **C.** Selected C parameter, **D.** Selected γ parameter.


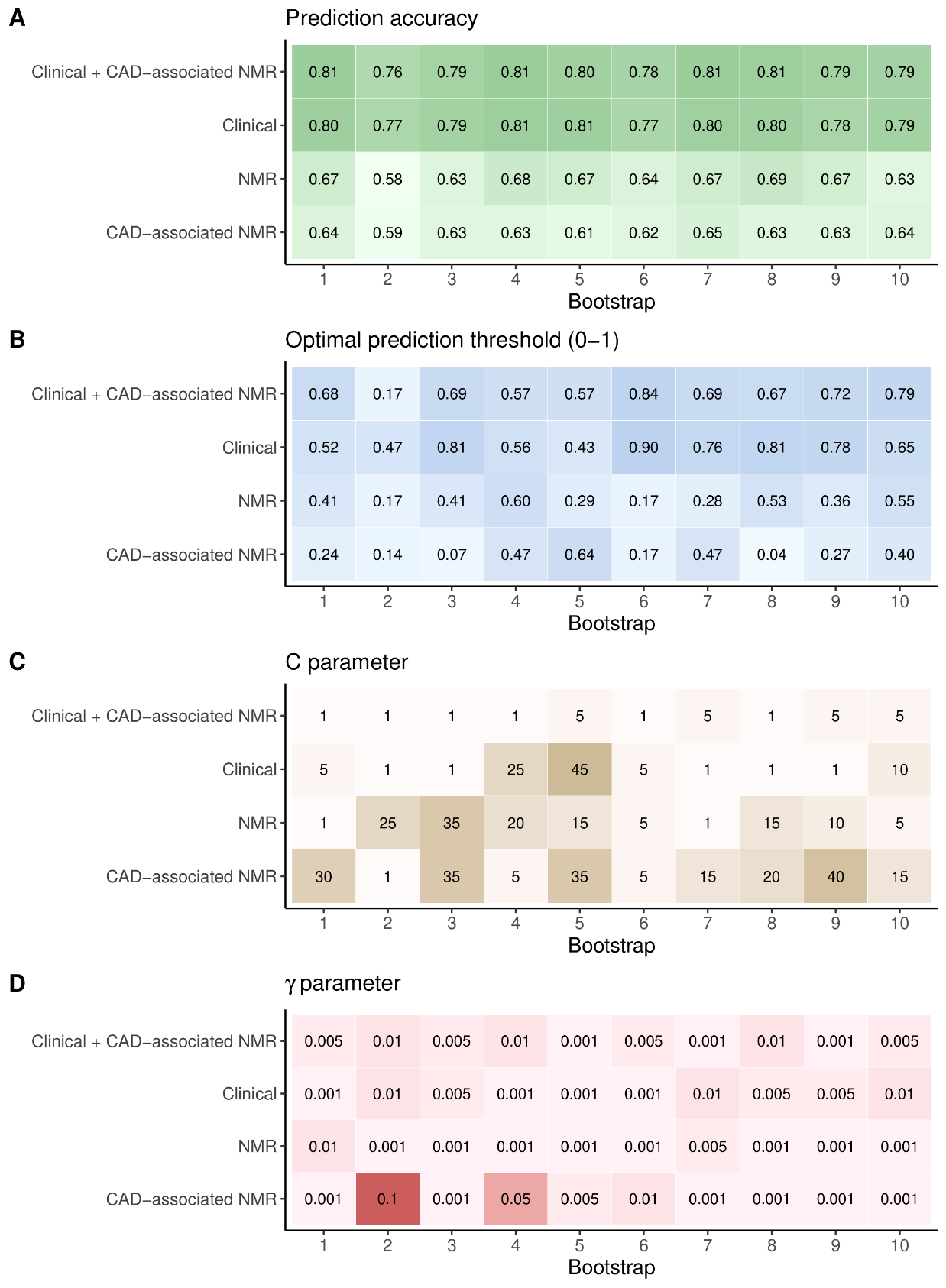


***CAD-associated NMR:*** Metabolites with missingness rate <15% and associated with incident CAD after full adjustment (*p*<0.05). ***NMR:*** Metabolites with missingness rate <15%. ***Clinical:*** sex, diabetes onset calendar year, diabetes onset age, age, diabetes duration, BMI, systolic blood pressure, diastolic blood pressure, mean arterial pressure, triglyceride, total cholesterol, HDL cholesterol, LDL cholesterol, HbA_1c_, eGFR, albuminuria.

**Fig. S16** 5-year coronary artery disease (CAD) prediction with support vector machine in each bootstrap. **A.** Prediction accuracy (AUC), **B.** Optimal prediction cutoff from the percentage score (0-1) with Youden method from ROC curve. **C.** Selected C parameter, **D.** Selected γ parameter.


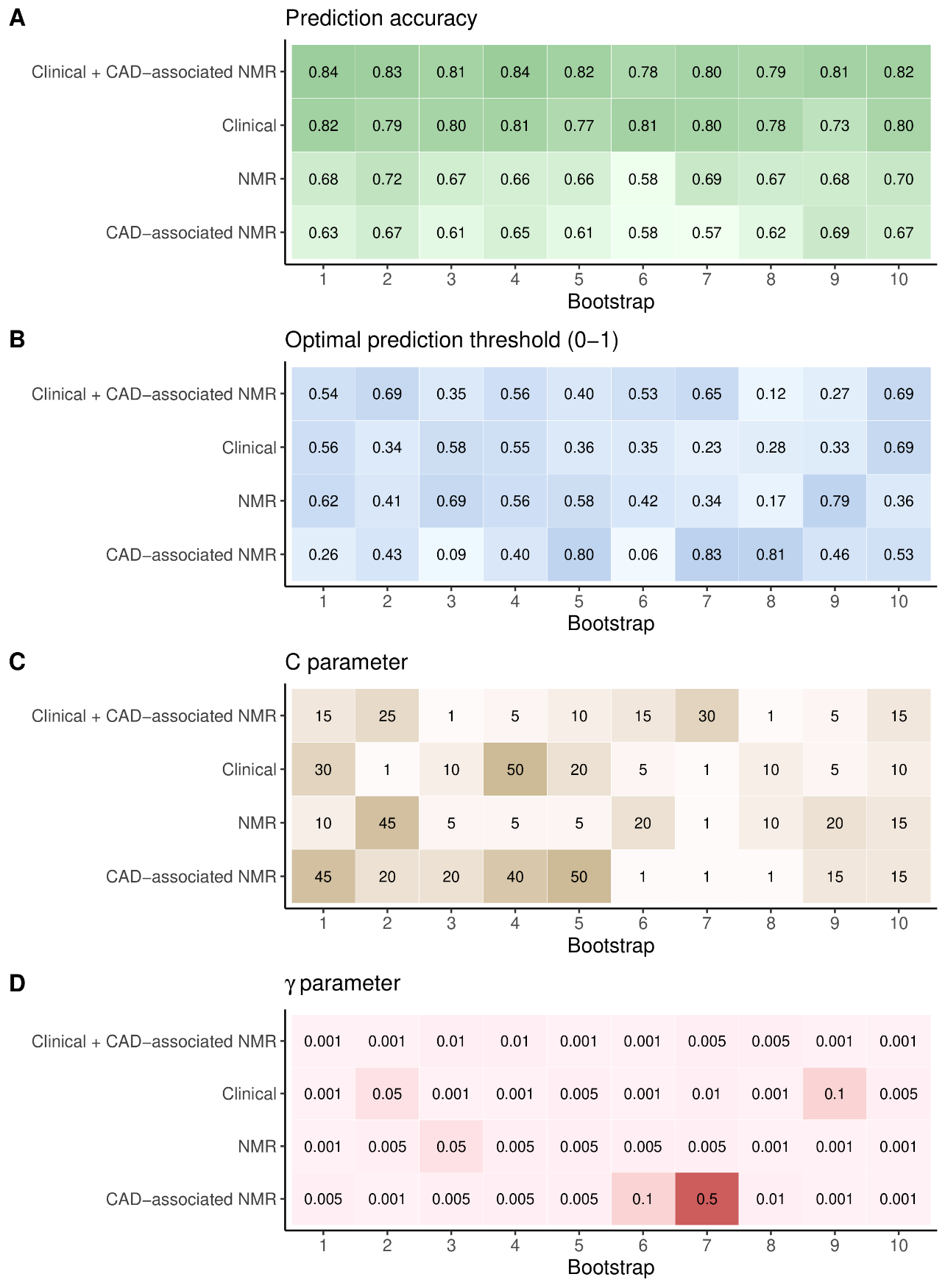


***CAD-associated NMR****:* Metabolites with missingness rate <15% and associated with incident CAD after full adjustment (p<0.05). ***NMR****:* Metabolites with missingness rate <15%. ***Clinical****:* sex, diabetes onset calendar year, diabetes onset age, age, diabetes duration, BMI, systolic blood pressure, diastolic blood pressure, mean arterial pressure, triglyceride, total cholesterol, HDL cholesterol, LDL cholesterol, HbA_1c_, eGFR, albuminuria.

# Supplementary Tables

## **Table S1** Coronary artery disease definition with registry data.

|  | Coronary artery disease | Milder events, excluded from controls |
| --- | --- | --- |
| 1. | **Myocardial infarction**  **ICD-10**: I21, I22, I23 **ICD-8 and ICD-9**: 410, 412 | **Angina pectoris** **ICD-10**: I20  **ICD-9**: 413 |
| 2. | **Coronary revascularization (*percutaneous coronary intervention or coronary artery bypass graft*)**  **Procedure codes**: FNE01, FNE02, FNE03, FNE10, FNE11, FNE20, FNE21, FNE96  FND10, FND20, FND96  FNB01, FNB02, FNB20, FNB96  FNA01, FNA02, FNA03, FNA04, FNA05, FNA10, FNA20, FNA96  FN1BT, FN1YT  TFN40, TFN50  FNC10, FNC20, FNC30, FNC40, FNC50, FNC60, FNC96  FNG  FNF  FN1AT  FN2 from 2016  **Procedure codes before 1996**: 5311, 5312, 5313, 5314, 5315, 5329  **ICD-10**: Z95.1, Z95.5 | **Other acute ischemic heart disease** **ICD-10**: I24  **ICD-9**: 411 |
| 3. |  | **Chronic ischemic heart disease** **ICD-10**: I25  **ICD-10**: 414 |
| 4. |  | **Heart failure** **ICD-10**: I50, I110, I130, I132  **ICD-9**: 4029B, 428  **ICD-8**: 4270, 7824 |

## **Table S2** Baseline clinical characteristics for cases who developed coronary artery disease (CAD) and controls who did not during the full follow-up (N=2,953, mean ± SD).

|  | Cases with incident CAD | Controls without incident CAD | *p*-value | Missing (N) |
| --- | --- | --- | --- | --- |
| N | 496 | 2,457 |  |  |
| Sex (male, %) | 251 (51%) | 1,201 (49%) | 0.52 |  |
| Diabetes onset calendar year^*^ | 1969 ± 13 | 1983 ± 16 | 5.62×10^-100^ |  |
| Age (year)^*^ | 46.95 ± 14.89 | 34.85 ± 15.84 | 5.27×10^-71^ |  |
| Diabetes duration (year)^*^ | 30.56 ± 13.70 | 17.79 ± 16.39 | 1.56×10^-82^ |  |
| Age at diabetes onset (year)^*^ | 14.16 ± 13.26 | 15.26 ± 13.64 | 0.059 |  |
| Estimated glomerular filtration rate (ml/min/1.73 m2)^*^ | 81 ± 37 | 99 ± 30 | 6.37×10^-46^ |  |
| HbA_1c_ (mmol/mol),  HbA_1c_ (%) | 71.63 ± 15.23,  8.70 ± 1.39 | 66.26 ± 15.36,  8.21 ± 1.41 | 5.06×10^-12^ | 144 |
| Systolic blood pressure (mmHg) | 141 ± 18 | 130 ± 16 | 3.04×10^-32^ | 301 |
| Diastolic blood pressure (mmHg) | 80 ± 10 | 79 ± 9 | 0.14 | 302 |
| Mean arterial pressure (mmHg) | 100 ± 11 | 96 ± 10 | 9.53×10^-15^ | 302 |
| Total cholesterol (mmol/l) | 5.26 ± 1.00 | 4.78 ± 0.87 | 2.07×10^-21^ | 6 |
| HDL cholesterol (mmol/l) | 1.30 ± 0.39 | 1.39 ± 0.39 | 1.17×10^-6^ | 7 |
| Triglyceride (mmol/l)^*^ | 1.12 ± 0.75 | 0.94 ± 0.59 | 1.40×10^-14^ | 6 |
| LDL cholesterol (mmol/l)^**^ | 3.36 ± 0.85 | 2.91 ± 0.81 | 8.03×10^-26^ | 7 |
| Body-mass-index (kg/m2) | 25.88 ± 3.56 | 25.16 ± 3.61 | 0.00010 | 320 |
| Waist-to-height ratio | 0.52 ± 0.06 | 0.50 ± 0.06 | 2.06×10^-16^ | 476 |
| Smoking (current or history, %) | 210 (49%) | 892 (43%) | 0.027 | 468 |
| Antihypertensive medication (yes, %) | 260 (59%) | 534 (25%) | 3.64×10^-43^ | 409 |
| Lipid-lowering medication (yes, %) | 97 (22%) | 150 (7%) | 2.68×10^-21^ | 408 |
| Albuminuria (yes, %) | 247 (50%) | 519 (21%) | 5.56×10^-40^ |  |

^*^Median ± IQR, ^**^LDL cholesterol calculated with Sampson formula.

## **Table S3** Urine metabolites. Detection limit, percent of missing values within the data (%), number of metabolite measurements replaced with detection limit (N), and distribution transformation method.

|  |  | 10-year CAD risk model data | | Life-time CAD risk model data | |  |
| --- | --- | --- | --- | --- | --- | --- |
|  | **Detection limit** | **Missing (%)** | **Replaced with detection limit (N)** | **Missing (%)** | **Replaced with detection limit (N)** | **Transformation** |
| Acetate | 5.85×10^-6^ | 0.48 | 24 | 0.51 | 33 | Square-root |
| Alanine | 0.0028 | 0.16 | 2 | 0.17 | 1 | Logarithmic |
| Allantoin | 0.023 | 34.39 | 0 | 35.08 | 0 | Logarithmic |
| 2-Hydroxyisobutyrate | 2.64×10^-4^ | 0.56 | 0 | 0.64 | 0 | Logarithmic |
| Arabinose | 0.023 | 7.40 | 0 | 7.70 | 0 | Logarithmic |
| 3-Aminoisobutyrate | 7.34×10^-6^ | 30.95 | 143 | 32.24 | 171 | Square-root |
| 3-Hydroxyisobutyrate | 5.64×10^-4^ | 0.40 | 6 | 0.34 | 4 | Logarithmic |
| 3-Hydroxyisovalerate | 4.60×10^-5^ | 0.08 | 10 | 0.07 | 11 | Logarithmic |
| cis-Aconitate | 0.0027 | 7.52 | 1 | 7.42 | 1 | Logarithmic |
| Citrate | 0.0010 | 3.40 | 16 | 3.18 | 14 | Square-root |
| Creatine | 6.32×10^-5^ | 52.50 | 4 | 51.24 | 4 | Square-root |
| Creatinine | 0.11 | 1.84 | 0 | 1.80 | 0 | Logarithmic |
| Dimethylamine | 1.32×10^-4^ | 0.84 | 12 | 0.85 | 14 | Square-root |
| 4-Deoxyerythronic acid | 4.48×10^-4^ | 7.68 | 0 | 7.28 | 0 | Logarithmic |
| 4-Deoxythreonate | 0.0036 | 9.72 | 0 | 8.97 | 0 | Logarithmic |
| Ethanolamine | 0.015 | 30.95 | 0 | 27.87 | 0 | Logarithmic |
| Ethanol | 0.0067 | 18.43 | 1 | 19.95 | 1 | Logarithmic |
| Formate | 1.71×10^-4^ | 0.40 | 10 | 0.37 | 7 | Logarithmic |
| 2-Furoylglycine | 7.08×10^-4^ | 4.48 | 2 | 5.01 | 0 | Square-root |
| Glucose | 0.0013 | 4.80 | 208 | 5.89 | 238 | Logarithmic |
| Glutamine | 0.030 | 5.52 | 0 | 5.66 | 0 | Logarithmic |
| Glycine | 0.0024 | 6.20 | 13 | 6.54 | 11 | Logarithmic |
| Glycolic acid | 0.026 | 42.62 | 0 | 40.64 | 0 | Logarithmic |
| Hippurate | 0.014 | 0 | 7 | 0 | 5 | Square-root |
| Histidine | 0.0084 | 41.18 | 0 | 40.33 | 0 | Logarithmic |
| HPHPA | 3.97×10^-4^ | 11.00 | 8 | 12.43 | 5 | Square-root |
| Hypoxanthine | 0.0025 | 14.23 | 0 | 14.77 | 0 | Logarithmic |
| Isoleucine | 0.0037 | 25.19 | 0 | 26.01 | 0 | Logarithmic |
| Indoxyl sulfate | 2.68×10^-5^ | 11.92 | 17 | 11.45 | 21 | Square-root |
| Lactate | 3.60×10^-5^ | 1.80 | 27 | 1.69 | 28 | Logarithmic |
| Leucine | 0.0023 | 0.88 | 0 | 0.85 | 0 | Logarithmic |
| Mannitol | 0.030 | 79.61 | 0 | 80.43 | 0 | Logarithmic |
| 3-hydroxyhippurate | 0.0077 | 5.08 | 1 | 5.01 | 0 | Logarithmic |
| 1-Methylnicotinamide | 1.15×10^-5^ | 0.04 | 186 | 0.03 | 224 | Square-root |
| Pyroglutamate | 0.0014 | 8.44 | 6 | 8.50 | 4 | Logarithmic |
| 4-Hydroxyhippurate | 6.19×10^-4^ | 1.60 | 1 | 1.46 | 1 | Logarithmic |
| Propylene glycol | 1.82×10^-5^ | 44.66 | 27 | 43.79 | 30 | Square-root |
| Proline betaine | 0.020 | 21.59 | 0 | 22.11 | 0 | Logarithmic |
| Pseudouridine | 0.0021 | 0.20 | 6 | 0.20 | 4 | Logarithmic |
| Quinic acid | 2.79×10^-4^ | 4.16 | 3 | 4.54 | 1 | Logarithmic |
| Sucrose | 5.16×10^-4^ | 3.76 | 2 | 3.83 | 2 | Logarithmic |
| trans-Aconitate | 1.12×10^-5^ | 4.16 | 1 | 4.44 | 1 | Logarithmic |
| Taurine | 0.034 | 85.01 | 0 | 84.93 | 0 | Logarithmic |
| Threonine | 6.06×10^-4^ | 1.36 | 1 | 1.29 | 1 | Logarithmic |
| Trimethylamine N-oxide (TMAO) | 4.18×10^-5^ | 1.28 | 110 | 1.73 | 123 | Square-root |
| 3-Methylhistidine | 0.020 | 38.63 | 0 | 39.69 | 0 | Logarithmic |
| Trigonelline | 3.15×10^-4^ | 0.04 | 35 | 0.03 | 54 | Square-root |
| Tryptophan | 0.0035 | 5.84 | 0 | 5.86 | 0 | Logarithmic |
| Tyrosine | 3.64×10^-4^ | 0.52 | 0 | 0.51 | 0 | Logarithmic |
| Uracil | 2.23×10^-4^ | 0 | 0 | 0 | 0 | Logarithmic |
| Urea | 0.63 | 0 | 6 | 0 | 4 | Logarithmic |
| Valine | 2.58×10^-4^ | 1.96 | 2 | 1.90 | 2 | Logarithmic |
| Xanthosine | 8.58×10^-4^ | 0 | 0 | 0 | 0 | Logarithmic |
| Xylose | 7.92×10^-4^ | 18.03 | 1 | 19.17 | 1 | Logarithmic |

## **Table S4** Baseline clinical characteristics for cases who developed coronary artery disease (CAD) and controls who did not, without (**A**) and with (**B**) albuminuria, during the 10-year follow-up (N=2,501, mean ± SD).

**A.**

|  | Cases with incident CAD | Controls without incident CAD | *p*-value | Missing (N) |
| --- | --- | --- | --- | --- |
| N | 103 | 1,619 |  |  |
| Sex (male, %) | 44 (43%) | 733 (45%) | 0.69 |  |
| Diabetes onset calendar year^*^ | 1966 ± 14 | 1981 ± 15 | 1.02×10^-20^ |  |
| Age (year)^*^ | 51.14 ± 11.59 | 39.36 ± 14.53 | 2.73×10^-18^ |  |
| Diabetes duration (year)^*^ | 33.84 ± 15.19 | 20.29 ± 15.08 | 1.68×10^-18^ |  |
| Age at diabetes onset (year)^*^ | 15.81 ± 12.77 | 17.84 ± 13.95 | 0.19 |  |
| Estimated glomerular filtration rate (ml/min/1.73 m2)^*^ | 91 ± 33 | 98 ± 28 | 1.93×10^-5^ |  |
| HbA_1c_ (mmol/mol),  HbA_1c_ (%) | 67.26 ± 13.49,  8.30 ± 1.23 | 64.69 ± 13.30,  8.07 ± 1.22 | 0.067 | 96 |
| Systolic blood pressure (mmHg) | 141 ± 18 | 131 ± 16 | 2.63×10^-7^ | 215 |
| Diastolic blood pressure (mmHg) | 77 ± 9 | 79 ± 9 | 0.11 | 215 |
| Mean arterial pressure (mmHg) | 99 ± 10 | 96 ± 10 | 0.019 | 215 |
| Total cholesterol (mmol/l) | 5.04 ± 0.93 | 4.84 ± 0.85 | 0.033 | 5 |
| HDL cholesterol (mmol/l) | 1.35 ± 0.36 | 1.43 ± 0.39 | 0.036 | 6 |
| Triglyceride (mmol/l)^*^ | 1.05 ± 0.58 | 0.89 ± 0.52 | 3.85×10^-5^ | 5 |
| LDL cholesterol (mmol/l)^**^ | 3.19 ± 0.87 | 2.96 ± 0.79 | 0.0099 | 6 |
| Body-mass-index (kg/m2) | 24.82 ± 2.91 | 25.33 ± 3.50 | 0.12 | 224 |
| Waist-to-height ratio | 0.51 ± 0.06 | 0.50 ± 0.06 | 0.055 | 317 |
| Smoking (current or history, %) | 36 (42%) | 549 (42%) | 1.00 | 315 |
| Antihypertensive medication (yes, %) | 36 (41%) | 218 (16%) | 7.47×10^-9^ | 279 |
| Lipid-lowering medication (yes, %) | 16 (18%) | 102 (8%) | 0.00086 | 279 |

^*^Median ± IQR, ^**^LDL cholesterol calculated with Sampson formula.

**B.**

|  | Cases with incident CAD | Controls without incident CAD | *p*-value | Missing (N) |
| --- | --- | --- | --- | --- |
| N | 106 | 673 |  |  |
| Sex (male, %) | 66 (62%) | 382 (57%) | 0.34 |  |
| Diabetes onset calendar year | 1967 ± 9 | 1973 ± 9 | 5.35×10^-10^ |  |
| Age (year)^*^ | 49.34 ± 14.65 | 38.91 ± 14.97 | 3.22×10^-16^ |  |
| Diabetes duration (year) | 33.06 ± 9.30 | 26.88 ± 8.87 | 2.34×10^-9^ |  |
| Age at diabetes onset (year)^*^ | 14.42 ± 14.77 | 11.91 ± 10.24 | 0.00038 |  |
| Estimated glomerular filtration rate (ml/min/1.73 m2)^*^ | 60 ± 39 | 82 ± 37 | 6.41×10^-11^ |  |
| HbA_1c_ (mmol/mol),  HbA_1c_ (%) | 75.20 ± 17.10,  9.03 ± 1.56 | 71.37 ± 15.33,  8.68 ± 1.40 | 0.034 | 31 |
| Systolic blood pressure (mmHg) | 149 ± 18 | 139 ± 18 | 1.88×10^-6^ | 47 |
| Diastolic blood pressure (mmHg) | 81 ± 12 | 82 ± 10 | 0.50 | 47 |
| Mean arterial pressure (mmHg) | 103 ± 11 | 101 ± 11 | 0.026 | 47 |
| Total cholesterol (mmol/l) | 5.56 ± 1.16 | 5.12 ± 0.94 | 0.00034 | 1 |
| HDL cholesterol (mmol/l) | 1.25 ± 0.41 | 1.33 ± 0.39 | 0.038 | 1 |
| Triglyceride (mmol/l)^*^ | 1.37 ± 1.03 | 1.11 ± 0.79 | 4.15×10^-5^ | 1 |
| LDL cholesterol (mmol/l)^**^ | 3.60 ± 0.93 | 3.21 ± 0.86 | 8.63×10^-5^ | 1 |
| Body-mass-index (kg/m2) | 26.55 ± 4.05 | 26.02 ± 3.81 | 0.24 | 54 |
| Waist-to-height ratio | 0.55 ± 0.07 | 0.52 ± 0.06 | 0.0021 | 83 |
| Smoking (yes/no) | 46 (49%) | 325 (54%) | 0.50 | 82 |
| Antihypertensive medication (yes/no) | 87 (93%) | 487 (79%) | 0.0039 | 72 |
| Lipid-lowering medication (yes/no) | 36 (38%) | 101 (16%) | 1.21×10^-6^ | 71 |

^*^Median ± IQR, ^**^LDL cholesterol calculated with Sampson formula.

**Table S5** 3-hydroxyisobutyrate (3-HIB) and estimated glucose disposal rate (eGDR). Linear regression between baseline eGDR and urinary 3-hydroxyisobutyrate. HbA_1c_, systolic blood pressure, and waist-to-height ratio are excluded from adjustment covariate setting, because eGDR formula includes HbA_1c_, systolic blood pressure, and central obesity.

| **Baseline eGDR ~ 3-HIB + adjustment covariates** | ***β*** | **SE** | ***p*-value** | **N** |
| --- | --- | --- | --- | --- |
| Non-modifiable | 0.0633 | 0.0456 | 0.17 | 2442 |
| Non-modifiable + kidney function | -0.186 | 0.0423 | 1.12×10^-5^ | 2442 |
| Non-modifiable + kidney function + LDL cholesterol + smoking | -0.171 | 0.0422 | 5.32×10^-5^ | 2392 |

Adjustment settings: **1.** Non-modifiable (age, sex, calendar year of diabetes onset), **2.** Non-modifiable + kidney function (eGFR, albuminuria), **C.** Non-modifiable risk factors + kidney function + LDL cholesterol + smoking.

**Table S6** 5-year coronary artery disease (CAD) risk models for metabolites displaying effect size time-dependency in the main analysis with a specific adjustment setting (N=2,501). No 5-year CAD risk specific control criteria was applied, thus, at the end of the follow-up, we had required age ≥30 years and duration ≥10 years for controls. Table showcase all considered 5-year CAD risk models.

**A.**

| **Adjustment setting** | **Metabolite** | **Proportional hazards *p*-value in 10-year model** | **HR [95% CI]** | ***p*-value** | **N  (CAD_yes/no_)** |
| --- | --- | --- | --- | --- | --- |
| Non-modifiable | 4-Deoxythreonate | 0.047 | 0.69 [0.55–0.89] | 0.0024 | 86/2157 |
|  | Tryptophan | 0.044 | 1.29 [1.07–1.57] | 0.0095 | 89/2229 |
|  | Urea | 0.045 | 1.19 [0.95–1.49] | 0.12 | 97/2358 |
| Non-modifiable + kidney function | Urea | 0.039 | 1.24 [0.99–1.56] | 0.062 | 97/2358 |
| Non-modifiable + kidney function + CAD risk factor | Allantoin | 0.026 | 0.92 [0.67–1.26] | 0.60 | 52/1217 |
|  | 4-Deoxythreonate | 0.016 | 0.60 [0.45–0.79] | 0.00032 | 70/1683 |
|  | Hypoxanthine | 0.034 | 0.79 [0.57–1.09] | 0.15 | 63/1576 |
|  | Leucine | 0.028 | 0.77 [0.61–0.98] | 0.037 | 78/1824 |
|  | trans-Aconitate | 0.019 | 0.66 [0.53–0.82] | 0.00014 | 74/1777 |

Adjustment settings: **1.** Non-modifiable (age, sex, calendar year of diabetes onset), **2.** Non-modifiable + kidney function (eGFR, albuminuria), **C.** Non-modifiable risk factors + kidney function + CAD risk factors (HbA_1c_, systolic blood pressure, LDL cholesterol, waist-to-height ratio, smoking).

**Table S7** 5-year coronary artery disease (CAD) risk models for urine metabolites displaying effect size time-dependency in the albuminuria status specific sub-analyses. No 5-year CAD risk specific control criteria was applied, thus, at the end of the follow-up, we had required age ≥30 years and duration ≥10 years for controls. Table showcase all considered 5-year CAD risk models for individuals with and without albuminuria (yes/no).

| **Albuminuria in participants (yes/no)** | **Metabolite** | **PH *p*-value in 10-year model** | **HR [95% CI]** | ***p*-value** | **N**  **(CAD_yes/no_)** |
| --- | --- | --- | --- | --- | --- |
| Yes | trans-Aconitate^*^ | 0.033 | 0.53 [0.39–0.71] | 3.09×10^-5^ | 42/567 |
| No | Allantoin | 0.024 | 0.86 [0.54–1.38] | 0.53 | 24/825 |
|  | 4-Deoxythreonate | 0.044 | 0.54 [0.35–0.83] | 0.0048 | 28/1167 |
|  | Hypoxanthine^*^ | 0.0029 | 0.55 [0.33–0.91] | 0.021 | 29/1112 |
|  | Isoleucine | 0.040 | 0.62 [0.37–1.04] | 0.071 | 25/890 |
|  | Leucine | 0.035 | 0.64 [0.44–0.92] | 0.017 | 34/1235 |
|  | Trimethylamine N-oxide^*^ | 0.0087 | 1.44 [1.12–1.85] | 0.0048 | 34/1231 |
|  | Uracil^*^ | 0.030 | 0.55 [0.37–0.81] | 0.0024 | 34/1248 |

Adjusted for age, sex, calendar year of diabetes onset, eGFR, HbA_1c_, systolic blood pressure, LDL cholesterol, waist-to-height ratio, smoking.

^*^Significant interaction effect with albuminuria on 5-year CAD risk (*p*<0.05).

## **Table S8** Global properties of the 10-year coronary artery disease data metabolic networks (N=2,501).

| **Model** | **Average degree** | **Average shortest path** | **Global clustering coefficient** |
| --- | --- | --- | --- |
| Complete data (N=2,501) | 34.69 | 1.51 | 0.64 |
| Individuals with incident CAD (N=209) | 7.41 | 3.04 | 0.54 |
| Correlation difference between individuals with and without incident CAD (N=209, N=2,292) | 5.42 | 2.79 | 0.20 |

**Table S9** Xanthosine and 3-hydroxyisobutyrate absolute concentration (mmol/l) association with clinical data. Adjusted for absolute urine creatinine concentration (mmol/l) in a regression model as an independent variable. Associations with significance below the network multiple testing correction significance threshold, i.e., link included to network (*p*<2×10^-5^), are bolded. Regression was performed with linear or logistic regression (stats package 4.3.0). Xanthosine and 3-hydroxyisobutyrate concentration are standardized to zero mean and unit variance.

|  | **Xanthosine (mmol/min)** | | | | **3-Hydroxyisobutyrate (mmol/min)** | | | |
| --- | --- | --- | --- | --- | --- | --- | --- | --- |
|  | ***β*** | **SE** | ***p*-value** | **N** | ***β*** | **SE** | ***p*-value** | **N** |
| Sex | **-0.53** | **0.11** | **1.81×10^-6^** | **2455** | -0.19 | 0.057 | 0.00081 | 2446 |
| Age (year) | 2.09 | 0.50 | 2.90×10^-5^ | 2455 | **-2.15** | **0.26** | **6.61×10^-16^** | **2446** |
| Diabetes duration (year) | **2.28** | **0.53** | **1.44×10^-5^** | **2455** | **-2.95** | **0.28** | **2.85×10^-26^** | **2446** |
| Diabetes onset calendar year | 1.10 | 0.54 | 0.042 | 2455 | **2.32** | **0.29** | **9.10×10^-16^** | **2446** |
| Diabetes onset age (year) | -0.19 | 0.46 | 0.68 | 2455 | 0.81 | 0.25 | 0.0011 | 2446 |
| eGFR (ml/min/1.73 m^2^) | 0.030 | 1.15 | 0.98 | 2455 | **9.00** | **0.59** | **8.25×10^-51^** | **2446** |
| Albuminuria (yes/no) | 0.065 | 0.11 | 0.55 | 2455 | **-0.75** | **0.069** | **6.11×10^-28^** | **2446** |
| HbA_1c_ (%) | **-0.47** | **0.066** | **3.06×10^-12^** | **2328** | **0.17** | **0.036** | **5.19×10^-6^** | **2320** |
| Systolic blood pressure (mmHg) | 1.80 | 0.91 | 0.047 | 2200 | **-2.10** | **0.48** | **1.44×10^-5^** | **2191** |
| Diastolic blood pressure (mmHg) | -1.80 | 0.50 | 0.00036 | 2200 | 0.56 | 0.27 | 0.038 | 2191 |
| Mean arterial pressure (mmHg) | -0.60 | 0.55 | 0.28 | 2200 | -0.33 | 0.29 | 0.27 | 2191 |
| LDL cholesterol (mmol/l) | -0.11 | 0.042 | 0.013 | 2448 | 0.015 | 0.023 | 0.51 | 2439 |
| Total cholesterol (mmol/l) | -0.047 | 0.046 | 0.31 | 2449 | 0.010 | 0.024 | 0.67 | 2440 |
| HDL cholesterol (mmol/l) | 0.068 | 0.020 | 0.00059 | 2448 | 0.0054 | 0.011 | 0.61 | 2439 |
| Triglyceride (mmol/l) | -0.0086 | 0.041 | 0.83 | 2449 | -0.021 | 0.022 | 0.34 | 2440 |
| BMI (kg/m^2^) | 0.15 | 0.19 | 0.44 | 2185 | 0.14 | 0.10 | 0.17 | 2176 |
| Waist-to-height ratio | 0.0040 | 0.0033 | 0.23 | 2065 | -0.0009 | 0.0018 | 0.62 | 2056 |
| Smoking (yes/no) | -0.057 | 0.11 | 0.60 | 2066 | 0.019 | 0.059 | 0.75 | 2058 |

## **Table S10** FinnDiane study centers.

| FinnDiane Study Centers | Physicians and nurses |
| --- | --- |
| Anjalankoski Health Center | S.Koivula, T.Uggeldahl |
| Central Finland Central Hospital, Jyväskylä | T.Forslund, A.Halonen, A.Koistinen, P.Koskiaho, M.Laukkanen, J.Saltevo, M.Tiihonen |
| Central Hospital of Åland Islands, Mariehamn | M.Forsen, H.Granlund, A.-C.Jonsson, B.Nyroos |
| Central Hospital of Kanta-Häme, Hämeenlinna | P.Kinnunen, A.Orvola, T.Salonen, A.Vähänen |
| Central Hospital of Kymenlaakso, Kotka | R.Paldanius, M.Riihelä, L.Ryysy |
| Central Hospital of Länsi-Pohja, Kemi | H.Laukkanen, P.Nyländen, A.Sademies |
| Central Ostrobothnian Hospital District, Kokkola | S.Anderson, B.Asplund, U.Byskata, P.Liedes, M.Kuusela, T.Virkkala |
| City of Espoo Health Center: |  |
| Espoonlahti | A.Nikkola, E.Ritola |
| Tapiola | M.Niska, H.Saarinen |
| Samaria | E.Oukko-Ruponen, T.Virtanen |
| Viherlaakso | A.Lyytinen |
| City of Helsinki Health Center: |  |
| Puistola | H.Kari, T.Simonen |
| Suutarila | A.Kaprio, J.Kärkkäinen, B.Rantaeskola |
| Töölö | P.Kääriäinen, J.Haaga, A-L.Pietiläinen |
| City of Hyvinkää Health Center | S.Klemetti, T.Nyandoto, E.Rontu, S.Satuli-Autere |
| City of Vantaa Health Center: |  |
| Korso | R.Toivonen, H.Virtanen |
| Länsimäki | R.Ahonen, M.Ivaska-Suomela, A.Jauhiainen |
| Martinlaakso | M.Laine, T.Pellonpää, R.Puranen |
| Myyrmäki | A.Airas, J.Laakso, K.Rautavaara |
| Rekola | M.Erola, E.Jatkola |
| Tikkurila | R.Lönnblad, A.Malm, J.Mäkelä, E.Rautamo |
| Heinola Health Center | P.Hentunen, J.Lagerstam |
| Helsinki University Hospital, Department of Medicine, Division of Nephrology | T.Claesson, A.Dufva, N.Elonen, M.Eriksson, J.Fagerudd, M.Feodoroff, D.Gordin, P.-H.Groop, O.Heikkilä, K.Hietala, S.Hägg-Holmberg, F.Jansson Sigfrids, M.Korolainen, J.Kytö, S.Lindh, H.Paajanen, K.Pettersson-Fernholm, K.Rimpeläinen, M.Rosengård-Bärlund, M.Rönnback, L.Salovaara, A.Sandelin, M.Saraheimo, S.Satuli-Autere, R.Simonsen, P.Smidtslund, L.Thorn, H.Tikkanen, J.Tuomikangas, A.Tynjälä, K.Uljala, T.Vesisenaho, J.Wadén, A.Ylinen |
| Herttoniemi Hospital, Helsinki | V.Sipilä |
| Hospital of Lounais-Häme, Forssa | T.Kalliomäki, J.Koskelainen, R.Nikkanen, N.Savolainen, H.Sulonen, E.Valtonen |
| Hyvinkää Hospital | L. Norvio, A.Hämäläinen |
| Iisalmi Hospital | E.Toivanen |
| Jokilaakso Hospital, Jämsä | A.Parta, I.Pirttiniemi |
| Jorvi Hospital, Helsinki University Central Hospital | S.Aranko, S.Ervasti, R.Kauppinen-Mäkelin, A.Kuusisto, T.Leppälä, K.Nikkilä, L.Pekkonen |
| Jyväskylä Health Center, Kyllö | K.Nuorva, M.Tiihonen |
| Kainuu Central Hospital, Kajaani | S.Jokelainen, K.Kananen, M.Karjalainen, P.Kemppainen, A-M.Mankinen, A.Reponen, M.Sankari |
| Kerava Health Center | H.Stuckey, P.Suominen |
| Kirkkonummi Health Center | A.Lappalainen, M.Liimatainen, J.Santaholma |
| Kivelä Hospital, Helsinki | A.Aimolahti, E.Huovinen |
| Koskela Hospital, Helsinki | V.Ilkka, M.Lehtimäki |
| Kotka Health Center | E.Pälikkö-Kontinen, A.Vanhanen |
| Kouvola Health Center | E.Koskinen, T.Siitonen |
| Kuopio University Hospital | E.Huttunen, R.Ikäheimo, P.Karhapää, P.Kekäläinen, M.Laakso, T.Lakka, E.Lampainen, L.Moilanen, S. Tanskanen, L.Niskanen, U.Tuovinen, I.Vauhkonen, E.Voutilainen |
| Kuusamo Health Center | T.Kääriäinen, E.Isopoussu |
| Kuusankoski Hospital | E.Kilkki, I.Koskinen, L.Riihelä |
| Laakso Hospital, Helsinki | T.Meriläinen, P.Poukka, R.Savolainen, N.Uhlenius |
| Lahti City Hospital | A.Mäkelä, M.Tanner |
| Lapland Central Hospital, Rovaniemi | L.Hyvärinen, K.Lampela, S.Pöykkö, T.Rompasaari, S.Severinkangas, T.Tulokas |
| Lappeenranta Health Center | P. Erola, L.Härkönen, P.Linkola, T.Pekkanen, I.Pulli, E.Repo |
| Lohja Hospital | T.Granlund, K.Hietanen, M.Porrassalmi, M.Saari, T.Salonen, M.Tiikkainen, |
| Länsi-Uusimaa Hospital, Tammisaari | I.-M.Jousmaa, J.Rinne |
| Loimaa Health Center | A.Mäkelä, P.Eloranta |
| Malmi Hospital, Helsinki | H.Lanki, S.Moilanen, M.Tilly-Kiesi |
| Mikkeli Central Hospital | A.Gynther, R.Manninen, P.Nironen, M.Salminen, T.Vänttinen |
| Mänttä Regional Hospital | I.Pirttiniemi, A-M.Hänninen |
| North Karelian Hospital, Joensuu | U-M.Henttula, P.Kekäläinen, M.Pietarinen, A.Rissanen, M.Voutilainen |
| Nurmijärvi Health Center | A.Burgos, K.Urtamo |
| Oulaskangas Hospital, Oulainen | E.Jokelainen, P-L.Jylkkä, E.Kaarlela, J.Vuolaspuro |
| Oulu Health Center | L.Hiltunen, R.Häkkinen, S.Keinänen-Kiukaanniemi |
| Oulu University Hospital | R.Ikäheimo |
| Päijät-Häme Central Hospital | H.Haapamäki, A.Helanterä, S.Hämäläinen, V.Ilvesmäki, H.Miettinen |
| Palokka Health Center | P.Sopanen, L.Welling |
| Pieksämäki Hospital | V.Sevtsenko, M.Tamminen |
| Pietarsaari Hospital | M-L.Holmbäck, B.Isomaa, L.Sarelin |
| Pori City Hospital | P.Ahonen, P.Merisalo, E.Muurinen, K.Sävelä |
| Porvoo Hospital | M.Kallio, B.Rask, S.Rämö |
| Raahe Hospital | A.Holma, M.Honkala, A.Tuomivaara, R.Vainionpää |
| Rauma Hospital | K.Laine, K.Saarinen, T.Salminen |
| Riihimäki Hospital | P.Aalto, E.Immonen, L.Juurinen |
| Salo Hospital | A.Alanko, J.Lapinleimu, P.Rautio, M.Virtanen |
| Satakunta Central Hospital, Pori | M.Asola, M.Juhola, P.Kunelius, M.-L.Lahdenmäki, P.Pääkkönen, M.Rautavirta |
| Savonlinna Central Hospital | T.Pulli, P.Sallinen, M.Taskinen, E.Tolvanen, T.Tuominen, H.Valtonen, A.Vartia, S-L.Viitanen |
| Seinäjoki Central Hospital | O.Antila, E.Korpi-Hyövälti, T.Latvala, E.Leijala, T.Leikkari, M.Punkari N.Rantamäki, H.Vähävuori |
| South Karelia Central Hospital, Lappeenranta | T.Ensala, E.Hussi, R.Härkönen, U.Nyholm, J.Toivanen |
| Tampere Health Center | A.Vaden, P.Alarotu, E.Kujansuu, H.Kirkkopelto-Jokinen, M.Helin, S.Gummerus, L.Calonius, T.Niskanen, T.Kaitala, T.Vatanen |
| Tampere University Hospital | P. Hannula, I.Ala-Houhala, R.Kannisto, T.Kuningas, P.Lampinen, M.Määttä,H.Oksala, T.Oksanen, A.Putila, H.Saha, K.Salonen, H.Tauriainen, S.Tulokas |
| Tiirismaa Health Center, Hollola | T.Kivelä, L.Petlin, L.Savolainen |
| Turku Health Center | A.Artukka, I.Hämäläinen, L.Lehtinen, E.Pyysalo, H.Virtamo, M.Viinikkala, M.Vähätalo |
| Turku University Central Hospital | K.Breitholz, R.Eskola, K.Metsärinne, U.Pietilä, P.Saarinen, R.Tuominen, S.Äyräpää |
| Vaajakoski Health Center | K.Mäkinen, P.Sopanen |
| Valkeakoski Regional Hospital | S.Ojanen, E.Valtonen, H.Ylönen, M.Rautiainen, T.Immonen |
| Vammala Regional Hospital | I.Isomäki, R.Kroneld, L.Mustaniemi, M.Tapiolinna-Mäkelä |
| Vasa Central Hospital | S.Bergkulla, U.Hautamäki, V-A.Myllyniemi, I.Rusk |
